# Engineering Synergistic Oxygen‐Proton Properties for High‐Performance Reversible Protonic Ceramic Cell Air Electrodes

**DOI:** 10.1002/smsc.202500256

**Published:** 2025-07-22

**Authors:** Na Yu, Xi Chen, Tong Liu, Shuo Zhai, Jiaxin Yuan, Yufei Song, Meng Ni

**Affiliations:** ^1^ Department of Building and Real Estate The Hong Kong Polytechnic University Hung Hom, Kowloon, Hong Kong P. R. China; ^2^ The Hong Kong Polytechnic University Shenzhen Research Institute Shenzhen 518057 Guangdong P. R. China; ^3^ State Key Laboratory of Intelligent Construction and Healthy Operation and Maintenance of Deep Underground Engineering Shenzhen University & Sichuan University Shenzhen 518060 P. R. China; ^4^ State Key Laboratory of Materials‐Oriented Chemical Engineering College of Chemical Engineering Nanjing Tech University Nanjing 210009 P. R. China

**Keywords:** air electrodes, balanced oxygen‐proton properties, reversible protonic ceramic cells, Ruddlesden–Popper perovskites, triple conductors

## Abstract

Reversible protonic ceramic cells (RePCCs) promise integration with renewable energy, supporting sustainable energy systems. RePCC performance hinges on the air electrode activity, where optimal proton, oxygen, and electron transport are essential. However, in air electrodes, oxygen exchange requires vacancies, while hydration consumes them, creating a fundamental trade‐off. Conventional material design strategies overemphasize hydration, overlooking their impact on oxygen transport. Here, using a simple Nb‐doped Sr_3_Fe_2_O_7–δ_ (SF) perovskite system, this study demonstrates that balanced oxygen–proton transport properties are essential for high‐performance air electrodes. Specifically, SF exhibits abundant oxygen vacancies, yet excessive hydration occupies these vacancies, thereby limiting oxygen‐ion transport and impairing oxygen electrocatalytic activity. Optimal Nb doping maintains the oxygen vacancy concentration while effectively suppressing excessive hydration due to the enhanced electrostatic repulsion between lattice cations and protons resulting from Nb doping. The resulting Sr_3_Fe_1.9_Nb_0.1_O_7–δ_ (SFNb0.1) electrode achieves a balance between oxygen and proton transport. Furthermore, Nb doping stabilizes the material's crystal structure. As a result, the electrode shows enhanced activity and stability. This work underscores balanced oxygen–proton transport as a key design principle for high‐performance RePCC air electrodes.

## Graphical abstract

Balanced oxygen‐proton properties are essential for achieving high‐performance air electrode materials for reversible protonic ceramic cells (RePCCs). This study rationally balances the oxygen‐proton properties of Ruddlesden–Popper‐type Fe‐based perovskite oxide through simple Nb doping, improving its oxygen catalysis activity. This work also offers a novel perspective for developing high‐performance air electrodes for RePCCs.

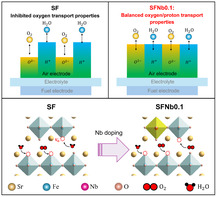

## 1 Introduction

The development and utilization of renewable energy necessitates the establishment of matching energy conversion and storage systems.^[^ ^1^, ^2^, ^3^, ^4^, ^5^ ^]^ Conventional batteries are limited by their linear cost‐capacity relationship, making them unsuitable for long‐term grid energy storage.^[^ ^6^ ^]^ In contrast, solid oxide cells (SOCs) can convert electrical energy into chemical energy stored in hydrogen, ammonia, or other fuels, thereby overcoming capacity limitations and enabling large‐scale, long‐duration energy storage, which makes them ideal electrochemical devices for renewable energy conversion.^[^ ^7^, ^8^, ^9^ ^]^ Additionally, SOCs can operate reversibly between fuel cell and electrolysis modes, enabling the construction of an efficient hydrogen–electricity cycle system with a round‐trip efficiency of up to 75%, suitable for diverse applications.^[^ ^10^, ^11^, ^12^ ^]^

SOCs typically require high‐temperature operation (700–1000 °C) to overcome the high energy barrier of oxygen‐ion conduction.^[^ ^13^ ^]^ However, high operating temperature accelerates component degradation and increases system costs. Reversible protonic ceramic cells (RePCCs), derived from SOCs, primarily involve proton conduction with a lower energy barrier, enabling operation at intermediate temperatures (350–650 °C),^[^ ^11^, ^14^ ^]^ which significantly enhances system stability while reducing cost and complexity. Furthermore, RePCCs produce purer electrolysis products because H_2_O is consumed at the air electrode, and dry, pure H_2_ can be generated at the fuel electrode, thereby lowering post‐processing costs.^[^ ^15^ ^]^

The performance of RePCCs critically depends on the activity of the air electrode, as the oxygen reduction reaction (ORR) and water oxidation reaction (WOR) kinetics are most sensitive to reduced operating temperatures. Efficient ORR/WOR activity relies on the synergistic transport of protons, oxygen ions, and electrons (H^+^/O^2−^/e^−^) within the air electrode.^[^ ^16^ ^]^ Given that RePCCs are derived from SOCs, their air electrode materials are often adapted from SOC air electrodes.^[^ ^17^, ^18^ ^]^ In SOCs, high‐performance mixed oxygen‐ion and electronic (O^2−^/e^−^) conductors are typically employed to meet ORR/OER requirements.^[^ ^19^, ^20^ ^]^ Thus, traditional RePCC air electrode research has focused on introducing proton conductivity into existing O^2−^/e^−^ mixed conductors to achieve the requisite H^+^/O^2−^/e^−^ triple conductivity. For example, Merkle et al. demonstrated through thermogravimetric (TG) analysis that adjusting cation composition significantly influences the hydration (proton uptake) properties of (Ba, Sr, La)(Fe, Co, Zn, Y)O_3−*δ*_ perovskite air electrodes, with low‐electronegativity Zn doping markedly enhancing hydration performance.^[^ ^21^ ^]^

Notably, in RePCC air electrodes, both oxygen and proton transport are mediated by oxygen vacancies: vacancies serve as the active sites for hydration and carriers for oxygen transport. This dual functionality creates a competitive relationship between the two processes: excessive hydration reduces oxygen vacancy concentration, thereby weakening oxygen surface exchange under humid conditions;^[^ ^22^ ^]^ conversely, insufficient hydration preserves vacancy concentration but limits proton conduction, as bulk proton migration relies on hopping between adjacent lattice oxygen sites.^[^ ^23^ ^]^ Therefore, achieving a balance between oxygen and proton transport properties is critical for optimizing electrochemical ORR/WOR activity. Despite this, current research on RePCC air electrodes overwhelmingly prioritizes enhancing proton conduction, with insufficient consideration given to balancing oxygen and proton transport properties.^[^ ^24^, ^25^ ^]^

This study demonstrates, using a simple Nb‐doped Sr_3_Fe_2_O_7‐*δ*_ (SF) perovskite system, that balanced oxygen–proton transport properties are essential to developing high‐performance air electrode materials. Specifically, SF exhibits abundant oxygen vacancies, yet excessive hydration occupies these vacancies, thereby limiting oxygen‐ion transport and impairing oxygen electrocatalytic activity. By optimizing Nb doping levels, the hydration properties and oxygen transport capability of SF perovskite are balanced. The optimized Sr_3_Fe_1.9_Nb_0.1_O_7‐*δ*_ (SFNb0.1) air electrode maintains the oxygen vacancy concentration while effectively suppressing excessive hydration due to enhanced electrostatic repulsion between the lattice cations and protons resulting from Nb doping, achieving a balanced proton/oxygen transport in the material. Additionally, Nb doping significantly improves structural stability, making SFNb0.1 an ideal RePCC air electrode material with both enhanced activity and durability. This study elucidates the critical role of balanced oxygen–proton transport in determining RePCC electrode performance and provides design principles for developing advanced triple conducting electrode materials.

## 2 Results and Discussion

### 2.1 Evolution of Material Structure, Composition, and Morphology

**Figure 1:**
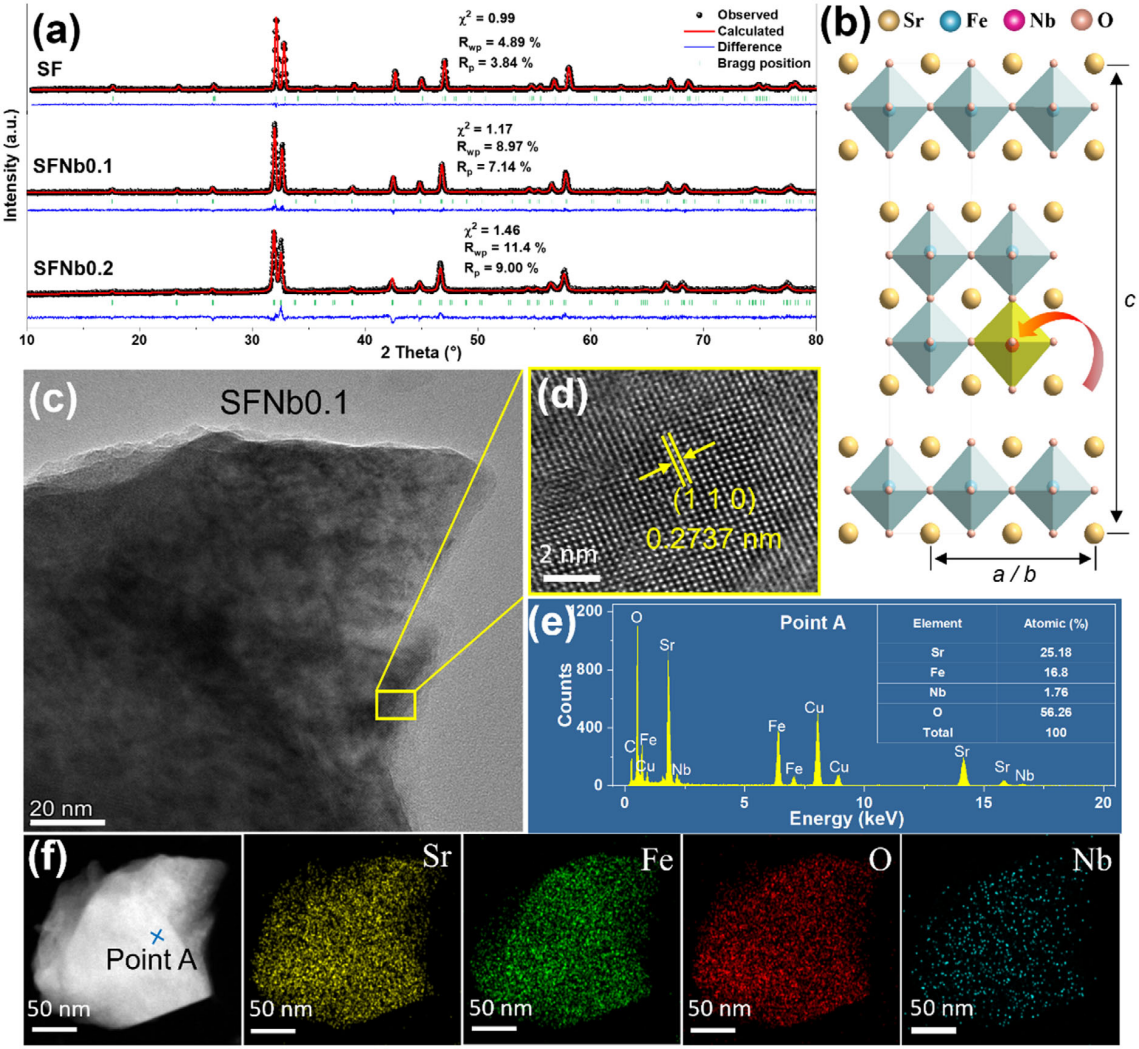
Phase structure of SFNbx. a) XRD refinement results of the fresh samples. b) Structural schematic diagram of SFNb0.1. c) TEM and d) HRTEM lattice fringes of SFNb0.1. e) EDX of SFNb0.1. f) HADDF‐EDX mapping results of SFNb0.1.

The SFNbx powders are synthesized via a sol‐gel method. After calcination at 1050 °C for 10 h, all three materials exhibit the Ruddlesden–Popper (RP) phase structure of Sr_3_Fe_2_O_6.74_ (PDF#01‐082‐0428), confirming successful phase formation (Figure S1, Supporting Information). For detailed structural analysis, Rietveld refinement of the X‐ray diffraction (XRD) patterns is performed (**Figure** **1** and Table S1, Supporting Information). The incorporation of Nb induces lattice expansion, as evidenced by increased unit cell parameters (a, b, c) and a corresponding volume increase from 0.301 nm^3^ (SF) to 0.306 nm^3^ (SFNb0.2). Figure 1b schematically presents the crystal structure of Nb‐doped Sr_3_Fe_2_O_7‐*δ*_.

The SFNb0.1 sample is selected for detailed microstructural characterization (Figure 1c). High‐resolution transmission electron microscopy (HRTEM) imaging (Figure 1d) reveals distinct lattice fringes corresponding to the (105) crystallographic planes. Energy‐dispersive X‐ray spectroscopy (EDX) analysis (Figure 1e) demonstrates elemental compositions of Sr (25.2%), Fe (16.8%), Nb (1.8%), and O (56.2%), which closely match the nominal stoichiometric ratios. high‐angle annular dark‐field (HAADF)‐scanning transmission electron microscopy coupled with EDX elemental mapping (Figure 1f) confirms uniform spatial distribution of all constituent elements within 200 nm SFNb0.1 particles, indicating excellent dopant incorporation and elemental homogeneity. Overall, these results confirm the successful incorporation of Nb ions into the B‐site of the parent SF lattice without inducing phase impurities or significant lattice distortion.

### 2.2 Mechanism of Balancing Oxygen‐Proton Properties

**Figure 2:**
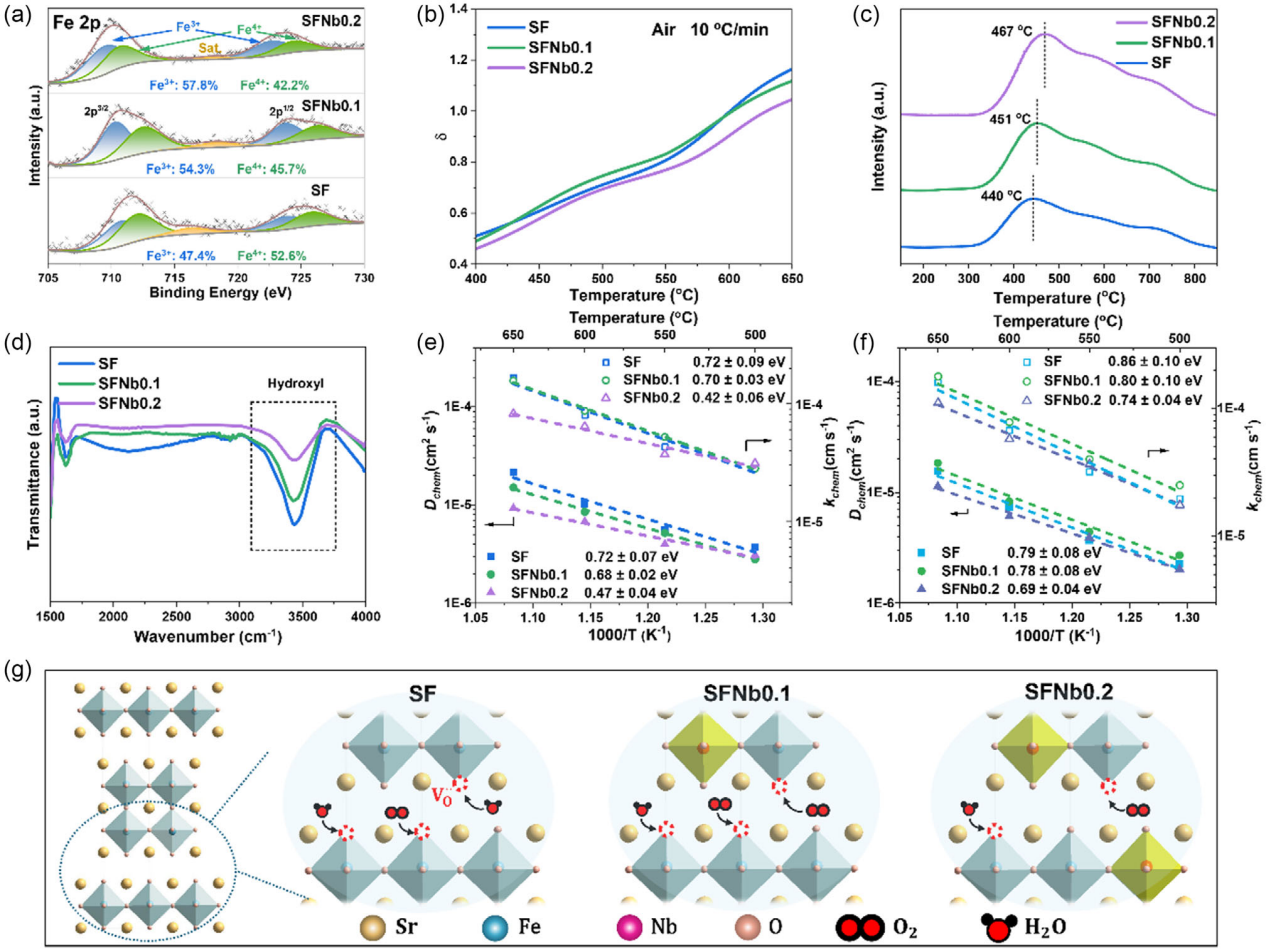
Intrinsic properties of SFNbx material. a) Fe 2p XPS spectra. b) Oxygen nonstoichiometry determined by iodometry and TG curves. c) O_2_–TPD curves. d) FTIR results of SFNbx samples after humid treatment for 1 h. Oxygen bulk diffusion coefficient (D_chem_) and surface exchange coefficient (*k*_chem_) in e) dry atmosphere and f) humid atmosphere. g) Mechanism of Nb‐tuning in air electrode performance.

Material surface properties significantly influence the electrode reaction activity. In this study, the surface elemental composition and valence states of fresh samples with different Nb doping levels are analyzed using X‐ray photoelectron spectroscopy (XPS), and the corresponding full spectra are presented in Figure S2, Supporting Information. Figure S3, Supporting Information, displays the Nb 3 d XPS spectra, where the peak intensity increases significantly with higher Nb doping levels. **Figure** **2**a shows the Fe 2p spectra of SFNbx materials, with peaks corresponding to Fe^3+^ located at about 710.1 and 723.6 eV, and those for Fe^4+^ at about 712.1 and 725.9 eV.^[^ ^26^, ^27^, ^28^ ^]^ As the Nb^5+^ doping level increases, the Fe^3+^ content continuously rises, while the Fe^4+^ content gradually decreases.

The surface oxygen nonstoichiometry (*δ*), calculated from the average valence states of the B‐site metal elements, is determined to be 0.47 for SF, 0.47 for SFNb0.1, and 0.42 for SFNb0.2. These results indicate that a low Nb doping level (SFNb0.1) does not alter the surface oxygen vacancy concentration, and the valence compensation is primarily achieved via reduction of the valence state of B‐site Fe ions. At higher Nb doping levels (SFNb0.2), the material maintains charge neutrality by reducing the average Fe valence and partially sacrificing oxygen vacancies.

The average bulk oxygen vacancy concentration of the three samples is determined using iodometric titration. With Nb incorporation, the average oxygen defect concentration decreases progressively, with values of 0.51, 0.49, and 0.46 for SF, SFNb0.1, and SFNb0.2, respectively. The discrepancy between the average *δ* and the surface *δ* indicates a greater bulk oxygen vacancy concentration than surface concentration in all materials. This originates from the unique layered structure of RP materials that facilitates rapid oxygen‐ion transport to the three‐phase boundary.^[^ ^29^, ^30^ ^]^ The bulk oxygen vacancy content in SF is higher than in SFNb0.1.

TG analysis (Figure S4, Supporting Information) shows all samples exhibit continuous weight loss from room temperature to 900 °C, with weight loss rates of 6.82%, 6.44%, and 6.17% for SF, SFNb0.1, and SFNb0.2, respectively. Weight loss below 200 °C is attributed to physically adsorbed water removal.^[^ ^31^ ^]^ At ≈400 °C, distinct slope changes indicate lattice oxygen release.^[^ ^32^, ^33^ ^]^ Oxygen loss quantification (Figure 2b) reveals SFNb0.1 maintains *δ* comparable to SF, while SFNb0.2 shows a significant reduction. The results clearly demonstrate that substitution with high‐valence Nb^5+^ ions effectively reduces oxygen vacancy concentration in the material through charge compensation mechanisms. Simultaneously, this substitution leads to a decreased average oxidation state of the substituted Fe^3+/4+^ redox couples. By precisely controlling the Nb doping concentration, the balance between these oxidation states can be finely tuned, enabling effective regulation of the oxygen vacancy environment. This optimized defect engineering may ultimately achieve an equilibrium between oxygen transport pathways and proton generation through hydration.

Oxygen desorption characteristics of the three samples are analyzed using O_2_‐TPD, with the results depicted in Figure 2c. The initial oxygen desorption temperatures for SF, SFNb0.1, and SFNb0.2 are 440, 451, and 467 °C, respectively. This indicates enhanced lattice stability through strengthened B‐site metal‐oxygen bonds by Nb doping.^[^ ^34^, ^35^ ^]^ The proton uptake behavior of SFNbx materials under humid conditions is investigated using Fourier‐transform infrared spectroscopy (FTIR).^[^ ^36^ ^]^ As shown in Figure 2d, the intensity of the –OH peak at 3500 cm^−1^ gradually decreases with increasing Nb doping content, indicating suppressed surface hydration.^[^ ^37^, ^38^ ^]^ The suppressed hydration capability of SFNb0.1, despite its similar surface oxygen vacancy concentration to SF, originates from the enhanced electrostatic repulsion effect induced by high‐valence Nb doping. As demonstrated by the hydration mechanism (Equation (1)) and the defect chemistry of RP‐type oxides,^[^ ^24^, ^39^, ^40^ ^]^ protons generated from water molecule dissociation must subsequently bond with lattice oxygen ions adjacent to oxygen vacancies.

(1) $$H_{2}O+V_{O}^{\cdot \cdot }+O_{O}^{\times }\to 2OH_{O}^{\cdot }$$

During this diffusion process, B‐site transition metal cations create significant electrostatic repulsion that hinders proton migration. Coulomb's law dictates that the electrostatic repulsion between charged ions scales with their charge states.^[^ ^41^ ^]^ The incorporation of Nb^5+^ introduces stronger Coulombic interactions compared to Fe^3+/4+^, thereby increasing the activation energy required for proton bonding. This effect leads to partial suppression of the proton generation process through hydration. Specifically, protons adsorbed on adjacent lattice oxygen sites experience significantly stronger repulsion from Nb^5+^ than from Fe cations, resulting in inhibited proton uptake.

To explore the oxygen transport properties, the electrical conductivity relaxation (ECR) curves of the three materials are measured at intermediate temperatures, as shown in Figure S5, Supporting Information. The ECR curves are obtained by stabilizing the oxygen partial pressure at 0.21 atm (21 vol% O_2_–79 vol% N_2_) and then rapidly switching it to 0.10 atm (10 vol% O_2_–90 vol% N_2_).

The ECRTOOLS is used to fit the curves and determine the surface oxygen exchange coefficient (*k*_chem_) and bulk oxygen diffusion coefficient (*D*_chem_) of the three samples,^[^ ^42^ ^]^ as shown in Figure 2e. As the testing temperature increases from 500 to 650 °C, the time required for the samples to reach a steady state significantly decreases, indicating enhanced bulk mass transport and surface exchange rates. When a small amount of Nb is introduced (SFNb0.1), the surface oxygen exchange performance of the material remains largely unchanged, while the bulk oxygen diffusion rate slightly decreases. As the Nb doping level increases to SFNb0.2, both *k*_chem_ and *D*_chem_ significantly decrease, which is likely due to the reduced surface and bulk oxygen vacancy content caused by excessive Nb doping.^[^ ^43^, ^44^ ^]^

Compared to the air electrode of SOCs, the electrochemical reactions at the air electrode of RePCCs are more complex, mainly due to the supply or generation of water vapor on the air electrode side during RePCC operation. Therefore, ECR tests are conducted in a humid atmosphere containing 3 vol% H_2_O (switching from 21% O_2_%–76% N_2_%–3% H_2_O to 10% O_2_%–87% N_2_%–3% H_2_O atmosphere) to explore the oxygen surface exchange and bulk diffusion performance of the materials under practical operating conditions, as shown in Figure S6, Supporting Information and Figure 2f. Compared to the dry atmosphere, the *k*_chem_ and *D*_chem_ of all three materials decrease under humid conditions, indicating competition between surface water/oxygen adsorption and bulk proton/oxygen transport. Additionally, the *D*_chem_ and *k*_chem_ of SFNb0.1 are slightly improved compared to SF, while the oxygen‐related performance of SFNb0.2 remains inferior to SF in both surface and bulk regions. These results suggest that a small amount of Nb doping enhances oxygen surface exchange and bulk transport performance under humid conditions, whereas excessive Nb doping adversely affects its oxygen‐related properties.

Based on the above findings, the mechanism by which Nb doping influences oxygen‐ and proton‐related properties is elucidated, as illustrated in Figure 2g. The incorporation of Nb simultaneously modulates oxygen vacancy characteristics and enhances surface electrostatic repulsion to suppress hydration, thereby regulating proton/oxygen transport and surface exchange kinetics. Crucially, SFNb0.1 achieves hydration suppression without altering oxygen vacancy concentration, enabling greater vacancy participation in oxygen exchange processes that synergistically enhances both surface reaction kinetics and bulk transport under humid conditions. In contrast, SFNb0.2 suffers from reduced surface/bulk oxygen vacancies that suppress oxygen transport and proton uptake. Consequently, SFNb0.1 exhibits optimally balanced oxygen/proton transport behavior, translating to superior electrocatalytic activity and operational stability.

### 2.3 Electrochemical Performance of Electrode Materials

**Figure 3:**
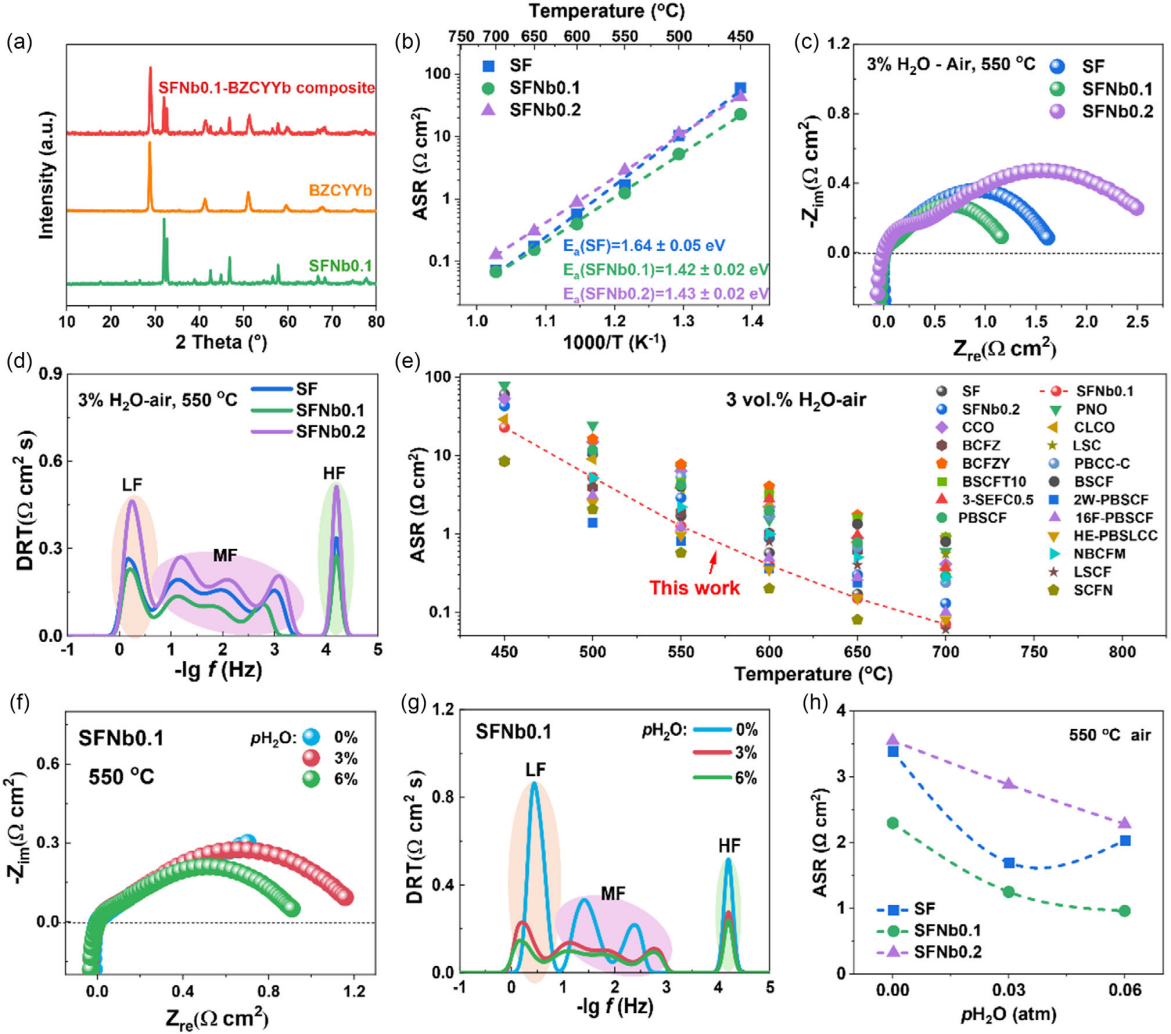
Electrochemical performance of SFNbx electrodes. a) Chemical compatibility between the BZCYYb electrolyte and SFNb0.1. b) Arrhenius plots of ASR for SFNbx electrodes at 450–700 °C. c) EIS spectra and d) DRT curves of SFNbx electrodes at 550 °C in 3 vol% H_2_O–air. e) Performance comparison of SFNbx electrodes with other reported advanced electrodes. f) EIS spectra and g) DRT curves of the SFNb0.1 electrode under different steam partial pressures. h) ASR variations of the three electrodes under different steam partial pressures.

The electrochemical performance of three candidate air electrodes was evaluated using symmetrical cells configured as SFNbx|BZCYYb|SFNbx. Prior to testing, the electrode powders are thoroughly mixed with the electrolyte material and calcined at 1100 °C for 5 h to examine the chemical compatibility between SFNbx and the electrolyte. The XRD patterns are shown in **Figure** **3**a. Only the characteristic XRD peaks of SFNb0.1 and BZCYYb were observed in the composite material, indicating chemical stability without interfacial reactions between components.

Subsequently, the polarization resistance (*R*_p_) or area‐specific resistance (ASR) of the three symmetrical cells under test conditions was obtained through electrochemical impedance spectra (EIS) testing. The Arrhenius plots of the ASR for the electrodes are shown in Figure 3b. In the temperature range of 450–700 °C under a 3 vol% H_2_O–air atmosphere, SFNb0.1 consistently exhibited the lowest *R*_p_, indicating enhanced ORR/WOR performance compared to the SF electrode, while the heavily doped SFNb0.2 showed a significant decline in performance. At 550 °C, the ASR values for the SF, SFNb0.1, and SFNb0.2 were 1.69, 1.25, and 2.88 Ω cm^2^, respectively (Figure 3c). Additionally, Nb doping reduced the activation energy (*E*_a_) of the electrode reactions, with *E*_a_ values for SF, SFNb0.1, and SFNb0.2 being 1.64, 1.42, and 1.43 eV, respectively.

To understand the detailed sub‐steps of the electrode reactions, the EIS curves under humid air at 550 °C were analyzed using DRTTOOLS,^[^ ^45^ ^]^ as shown in Figure 3d. The peak areas represent the *R*_p_ values of the corresponding electrochemical sub‐processes. Additionally, these characteristic peaks can be categorized into three frequency domains: 1) low‐frequency (LF, 10^−1^–10^1^ Hz) corresponding to gas diffusion and surface adsorption; 2) mid‐frequency (MF, 10^1^–10^4^ Hz) associated with surface exchange and bulk transport; and 3) high‐frequency (HF, >10^4^ Hz) related to ion transport.^[^ ^45^, ^46^ ^]^ The MF peaks demonstrate the largest spectral area across all electrodes (Figure S7, Supporting Information), indicating that surface exchange and bulk transport constitute the rate‐limiting steps for electrode reactions. Notably, SFNb0.2 exhibits significantly enlarged LF and MF peak areas relative to SF, suggesting impaired gas diffusion/surface adsorption and surface exchange/bulk transport processes. This degradation likely stems from reduced oxygen vacancy concentrations, where diminished surface vacancies compromise O_2_/H_2_O adsorption sites, while insufficient bulk vacancies hinder O^2−^ diffusion. Conversely, SFNb0.1 shows substantially reduced MF resistance versus SF, reflecting accelerated ion generation and transport kinetics. This observation aligns with findings shown in Figure 2, confirming that SFNb0.1 achieves optimal hydration/proton conductivity–oxygen exchange/diffusion equilibrium under humid conditions, thereby enabling superior oxygen/proton cotransport kinetics.

The performance of the SFNbx electrodes was compared with other advanced air electrodes reported in the literature under the same test conditions (Figure 3e and Table S2, Supporting Information). The results show that SFNb0.1 exhibits outstanding performance among Fe‐based electrodes, even surpassing some advanced Co‐based electrodes such as Ba_0.5_Sr_0.5_Co_0.8_Fe_0.2_O_3−*δ*_ (BSCF), BaCo_0.4_Fe_0.4_Zr_0.1_Y_0.1_O_3−*δ*_ (BCFZY), and PrBa_0.5_Sr_0.5_Co_1.5_Fe_0.5_O_5+*δ*_ (PBSCF).^[^ ^47^, ^48^, ^49^ ^]^

Furthermore, the electrochemical performance of the three electrodes under different steam pressures was analyzed, as shown in Figure 3f and Figure S8, Supporting Information. Compared to a dry atmosphere, the introduction of 3% vapor significantly improves the performance of all three electrodes, with a notable reduction in *R*_p_. Distribution of relaxation time (DRT) analysis reveals that with the introduction of vapor, the LF and MF resistances decrease significantly, indicating substantial optimization of gas adsorption, surface exchange, and bulk diffusion processes on the electrode surface. This improvement is primarily attributed to the hydration reaction of SFNbx materials in a humid atmosphere, leading to the formation and transport of proton defects that extends the reaction sites from the original electrode/electrolyte interface to the entire electrode.^[^ ^16^ ^]^ Consequently, the SFNbx electrodes have significant potential for superior electrocatalytic performance.

The ASR of the three electrodes at 550 °C under different steam pressures was further quantified (Figure 3h and Figure S9, Supporting Information). With increasing steam partial pressure, the *R*_p_ of SFNb0.1 and SFNb0.2 continues to decrease, while the SF electrode exhibits the lowest value at 3% water vapor, indicating that the SF electrode has poorer tolerance to higher steam pressures. This may be related to the higher bulk oxygen vacancy content in the SF electrode. According to the Grotthuss mechanism, which describes proton transport in perovskites, excessive bulk oxygen defects increase proton transport resistance.^[^ ^23^, ^50^, ^51^ ^]^ Furthermore, the increased proton migration resistance may impede the liberation of oxygen vacancies from surface hydration reactions, thereby inhibiting oxygen adsorption and dissociation processes at the electrode surface.^[^ ^52^ ^]^ Collectively, these findings reaffirm that achieving balanced oxygen‐ion and proton transport behavior is critical for optimizing the electrochemical performance of air electrodes under a humid atmosphere.

### 2.4 RePCC Application

**Figure 4:**
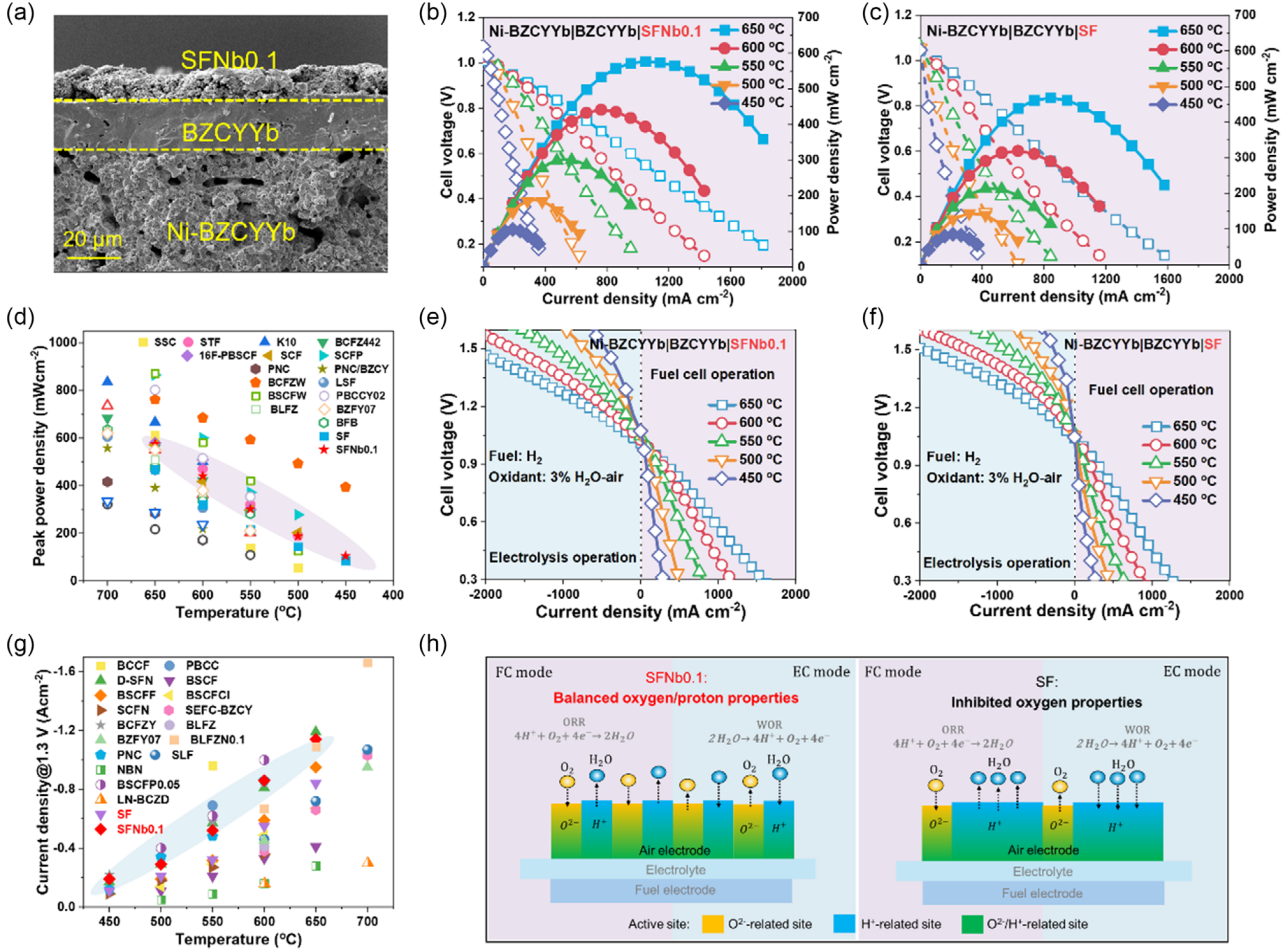
Electrochemical performance and microstructure of single cells. a) Cross‐sectional SEM of the SFNb0.1‐based cell after testing. Single cell power density curves with different air electrodes: b) SFNb0.1, c) SF. d) Maximum power density compared to the performance reported in the literature. Electrolysis polarization curves with different air electrodes: e) SFNb0.1, f) SF. g) Comparison of current densities of the SFNb0.1 electrode with other advanced air electrodes reported recently. h) Schematic diagrams of working mechanisms for SFNb0.1 and SF air electrodes.

To assess the ORR/WOR activity of SFNb0.1 as an air electrode, a single cell configured as Ni‐BZCYYb|BZCYYb|SFNb0.1 is employed. **Figure** **4**a displays the cross‐sectional view of the single cell post‐testing, where the SFNb0.1 electrode is observed to have excellent contact with the dense BZCYYb electrolyte with a thickness of ≈17 μm. During testing, the Ni–BZCYYb fuel electrode is supplied with 50 sccm of dry H_2_, while the SFNb0.1 air electrode is exposed to 100 sccm of 3 vol% H_2_O–air atmosphere. Figure 4b illustrates the power density curves of the single cell with the SFNb0.1 air electrode at temperatures ranging from 450 to 650 °C. The maximum power densities achieved at 650, 600, 550, 500, and 450 °C are 576, 442, 302, 188, and 105 mW cm^−2^, respectively. For comparison, a single cell with an SF air electrode is also fabricated, maintaining a configuration similar to that of the SFNb0.1 air electrode cell, except for the air electrode material (as shown in Figure S10, Supporting Information). As depicted in Figure 4c, the power output of the cell with the SF air electrode is significantly lower than that of the SFNb0.1 air electrode cell across the temperature range of 650–450 °C, with maximum power densities of 468, 319, 214, 144, and 85 mW cm^−2^ at the respective temperatures. Given the identical configurations of both cells, the performance discrepancy is primarily attributed to the difference in air electrode performance. The SFNb0.1 air electrode markedly enhances the output performance of the single cell.

Literature‐reported advanced protonic ceramic fuel cell (PCFC) performances under similar testing conditions are summarized and presented in Figure 4d and Table S3, Supporting Information. The electrochemical performance of the SFNb0.1 air electrode demonstrates competitive characteristics, surpassing most Fe‐based air electrodes and even some Co‐based counterparts.^[^ ^53^, ^54^, ^55^, ^56^ ^]^ It should be noted that the performance of single cells significantly improves when the electrolyte thickness is reduced. Therefore, the performance of the SFNb0.1 air electrode‐based single cell can be further optimized by refining the electrolyte configuration.

To investigate the performance of the air electrode in different modes, the polarization curves of the cell in both fuel cell (FC) and electrolysis cell (EC) modes are further examined (Figure 4e). At 1.3 V, the current densities of the SFNb0.1 air electrode‐based cell at 650, 600, 550, and 500 °C are −1135, −793, −512, and −302 mA cm^−2^, respectively, which are significantly higher than those of the SF air electrode‐based cell (−842, −552, −336, and −187 mA cm^−2^) (Figure 4f). As shown in Figure 4g and Table S4, Supporting Information, compared to advanced Fe‐based air electrodes such as Sr_0.9_Ce_0.1_Fe_0.8_Ni_0.2_O_3−*δ*_ and Sr_2.8_La_0.2_Fe_2_O_7−*δ*_ or Co‐based air electrodes like Ba_0.5_Sr_0.5_Co_0.8_Fe_0.2_O_3−*δ*_ and BaCo_0.4_Fe_0.4_Zr_0.1_Y_0.1_O_3−*δ*_, the SFNb0.1 air electrode demonstrates competitive electrolysis performance.^[^ ^16^, ^43^, ^57^ ^]^

The mechanism by which the SFNb0.1 air electrode enhances cell performance in both fuel cell and electrolysis modes is illustrated in Figure 4h. In FC mode, a balanced proton formation and oxygen surface adsorption facilitate the extension of the ORR reaction across the entire electrode surface for the SFNb0.1 air electrode, whereas excess surface hydration reactions hinder oxygen adsorption on the SF electrode surface and impede subsequent oxygen dissociation/reduction and diffusion. In EC mode, balanced proton/oxygen transport properties aid in the adsorption of water molecules and the release of oxygen for the SFNb0.1 air electrode, thereby promoting WOR and the electrolysis process. Conversely, excessive water surface adsorption makes it difficult for oxygen vacancies on the SF electrode surface to be released, obstructing oxygen formation and desorption, and thus affecting electrolysis performance. Therefore, the balanced proton and oxygen transport properties enable the SFNb0.1 air electrode to exhibit superior ORR/WOR performance in both FC and EC modes.

### 2.5 Stability Evaluation

**Figure 5:**
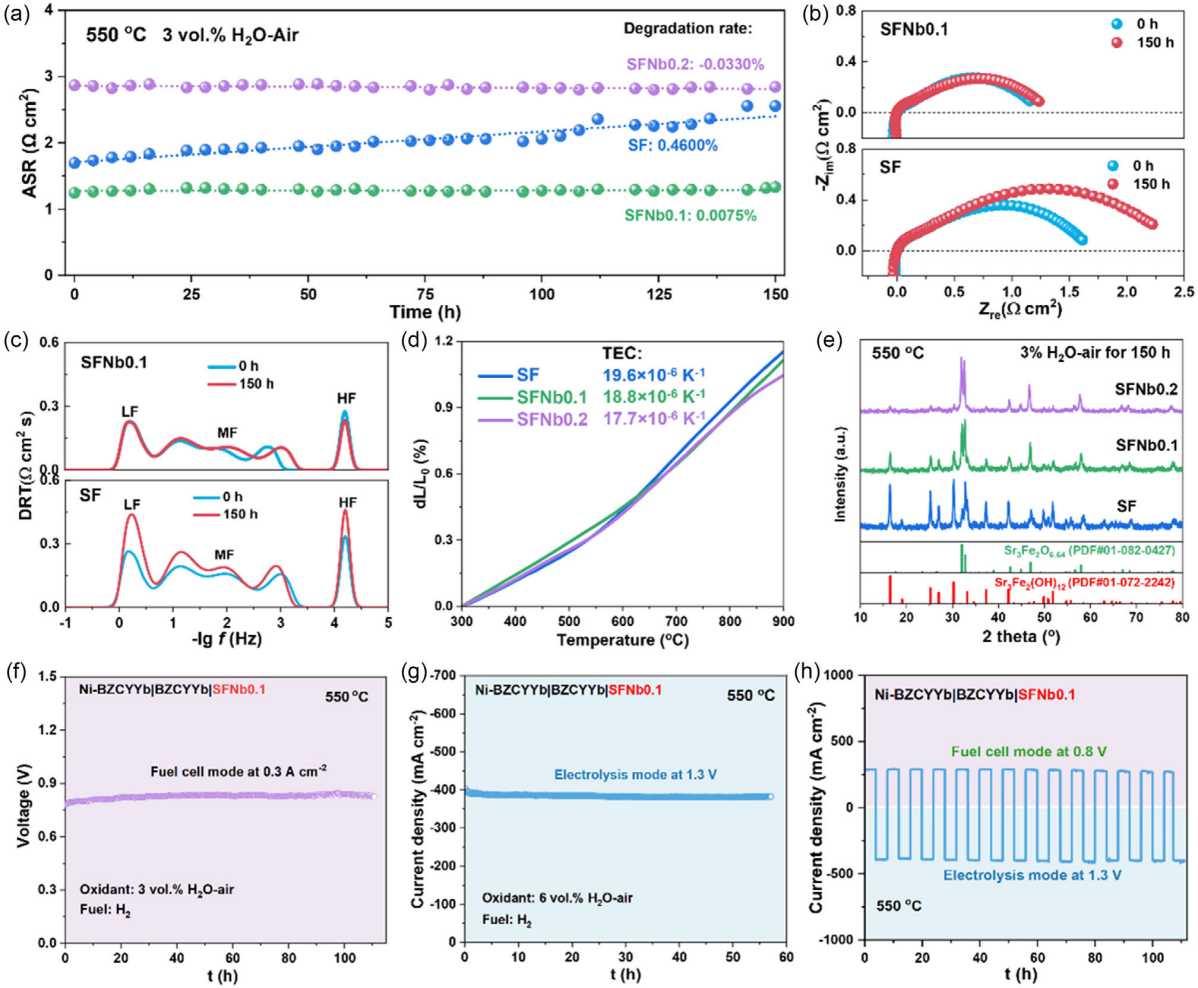
Stability tests of SFNbx electrodes. a) Symmetrical cell stability results in 3 vol% H_2_O–air. b) EIS curves and c) DRT plots before and after the stability test. d) Thermal expansion behavior during 300–900 °C. e) XRD patterns of SFNbx after 150 h wet treatment. f) Stability test of SFNb0.1‐based PCFC at 0.3 A cm^−2^. g) Durability test of SFNb0.1‐based PCEC at 1.3 V. h) Long‐term test of RePCC based on SFNb0.1 air electrode.

In addition to possessing superior electrochemical properties, air electrodes are also required to demonstrate satisfactory operational stability. Initially, the long‐term performance evolution of these three electrodes in a humid atmosphere over 150 h is evaluated through symmetrical cell testing, as illustrated in **Figure** **5**a. The degradation rate of the SF electrode is measured at 0.46% h^−1^, whereas the degradation rate of SFNb0.1 is significantly reduced to 0.0075% h^−1^, with SFNb0.2 showing almost no performance degradation. To elucidate the reasons for significantly enhanced electrode kinetics, EIS spectra and corresponding DRT analysis of the three electrodes before and after the long‐term testing are presented in Figure 5b, c, and Figure S11, Supporting Information. A significant increase in the resistances associated with LF and MF processes is observed for the SF electrode after 150 h‐operation, while the SFNb air electrode exhibits virtually unchanged characteristics, demonstrating enhanced resistance to degradation in surface adsorption, surface exchange, and bulk transport sub‐processes. The incorporation of Nb is found to significantly enhance the long‐term stability of the electrode performance, particularly in terms of surface exchange and bulk diffusion processes.

Furthermore, the thermochemical stability of the three electrodes is evaluated using a thermal expansion tester, as depicted in Figure 5d. The incorporation of Nb partially reduces the average linear thermal expansion coefficients (TECs) of SFNbx, decreasing from 19.6 × 10^−6^ K^−1^ for SF to 18.8 × 10^−6^ K^−1^ for SFNb0.1 and 17.7 × 10^−6^ K^−1^ for SFNb0.2. Additionally, the TECs of Nb‐doped SFNbx are significantly lower than those of state‐of‐the‐art Co‐based air electrodes, such as LaBa_0.5_Sr_0.5_Co_2_O_5+*δ*_ (26.2 × 10^−6^ K^−1^), and BaCoNbO_3–*δ*_ (24.2 × 10^−6^ K^−1^) (shown in Table S5, Supporting Information).^[^ ^58^, ^59^ ^]^ The reduced TECs are also beneficial for the long‐term stable operation of the electrodes, as the TEC of the BZCYYb electrolyte is typically around 10 × 10^−6^ K^−1^.^[^ ^60^ ^]^ The lowered TECs help alleviate interfacial stress between the air electrode and electrolyte during intermediate‐temperature operation, thereby enhancing the system's long‐term operational stability.

Additionally, the phase composition changes of the three powders under the long‐term operating conditions of the cell (550 °C, 3% H_2_O–air) are examined, as shown in Figure 5e. The phase composition is further determined through XRD refinement (Figure S12, Supporting Information). Significant phase transformation is observed in SF after 150 h‐operation, while the incorporation of Nb effectively suppresses the formation of secondary phases, improving the phase structural stability of the material in a humid atmosphere, which represents a crucial factor for maintaining high ORR/OER catalytic activity. These results indicate that the markedly enhanced electrode kinetic stability primarily stems from improved thermal compatibility and hydration tolerance, which enable robust electrode/electrolyte interfaces and stabilized catalytic phases. Furthermore, combined with the analysis results from Figure 1 and 2, this improvement may originate from stronger Nb—O bonding interactions and enhanced electrostatic repulsion toward protons. These effects not only directly reinforce the material structure but also substantially suppress the over hydration process of the parent lattice.

Moreover, the stability of the SFNb0.1 air electrode is assessed based on single‐cell tests. As shown in Figure 5f, the single cell demonstrates a stable power output for ≈110 h at a current density of 0.3 A cm^−2^. The initial voltage increase during the early testing phase may be attributed to the electrode activation process.^[^ ^61^, ^62^ ^]^ Additionally, the durability of the single cell with SFNb0.1 electrode in electrolysis mode is examined, as presented in Figure 5g. The cell demonstrates stable performance with slight degradation during a 57 h electrolysis test at 1.3 V. The robust interface constructed between the air electrode and electrolyte (Figure 4a) represents one of the crucial factors enabling stable cell operation. Finally, the cell exhibits stable output performance during cyclic operation for nearly 112 h in both FC mode (at 0.8 V) and EC mode (at 1.3 V), confirming its excellent reversibility (Figure 5h).

## 3 Conclusion

In this work, the synergistic regulation of oxygen and proton transport properties in SF‐based air electrode materials is successfully achieved through controlled Nb^5+^ ion incorporation. The optimized SFNb0.1 material maintains its oxygen vacancy concentration while effectively suppressing excessive hydration through Nb‐doping‐enhanced electrostatic repulsion between lattice cations and protons, ultimately realizing a balance between proton and oxygen transport within the material. At 650 °C, the RePCC based on the SFNb0.1 air electrode demonstrates a maximum power density of 576 mW cm^−2^ in PCFC mode and achieves a current density of −1.14 A cm^−2^ at 1.3 V in PCEC mode, showing superior performance compared to the pristine SF electrodes. This result confirms the effectiveness of balanced proton and oxygen transport properties in enhancing the ORR/WOR performance of air electrodes. Furthermore, the introduction of Nb enhances both structural stability and thermal stability of the material. During a 112‐hour test, the RePCC with SFNb0.1 air electrode exhibits outstanding durability. This research elucidates the critical role of balanced oxygen–proton transport in determining the performance of RePCC air electrodes and establishes design principles for developing advanced electrode materials.

## 4 Experimental Section

4.1

4.1.1

##### Material Synthesis

SFNbx (*x* = 0, 0.1, 0.2) were prepared by the sol‐gel method.^[^ ^43^, ^63^ ^]^ Stoichiometric amounts of Sr(NO_3_)_2_ (AR, 99.5%), Fe(NO_3_)_3_·9H_2_O (AR, 99.5%), and C_10_H_5_NbO_20_ (AR, 99%) were weighed and dissolved in deionized water. Citric acid (AR, 99.5%) and EDTA (AR, 99.5%) were added as complexing agents and fuels in a molar ratio of 1.5:1 and 1:1 with the metal ions, respectively. Ammonia water (AR, 26%–28%) was used to adjust the solution pH to 7–8. The solution was then heated to reduce the solvent and form a gel, which was subsequently transferred to an oven at 180 °C to obtain the precursor. The collected powder was calcined at 1100 °C for 10 h to obtain the initial SFNbx powders.

##### Material Characterization

The phase structure of the materials was analyzed using X‐ray diffraction (XRD, Rigaku SmartLab 9 kW). X‐ray photoelectron spectroscopy (XPS, Thermo Fisher Scientific Nexsa) was employed to detect the surface elemental composition of the materials. Scanning electron microscopy (SEM, Tescan MIRA) was used to observe the microstructure of the cells after testing. Transmission electron microscopy (HRTEM, FEI Talos F200x) was utilized to examine the microscopic structure of the sample surfaces. Energy‐dispersive X‐ray spectroscopy (EDX, FEI Talos F200x) and high‐angle annular dark‐field (HAADF)‐EDX mapping were applied to assess the elemental distribution within the materials. The iodometric titration method was employed to determine the oxygen vacancy concentration of the samples at room temperature, with experimental details referred to in previous research.^[^ ^22^ ^]^ The oxygen vacancy concentration of the SFNbx samples at room temperature was obtained by averaging the results from three parallel sample tests. Thermogravimetric analysis (TG, TGA5500) was employed to measure lattice oxygen loss at high temperatures. Fourier‐transform infrared spectroscopy (FTIR, Thermo Scientific Nicolet iS5) was used to evaluate the hydration properties of the materials under humid conditions. O_2_–temperature‐programmed desorption (O_2_‐TPD, TP5080B) was carried out to investigate the activity of lattice oxygen for SFNbx samples. The surface oxygen adsorption and bulk oxygen diffusion behaviors of SFNbx samples were investigated using ECR tests, where voltage–current signals were recorded by a digital source meter (Keithley 2440), with detailed testing methods described in previous research.^[^ ^22^, ^43^ ^]^ The TEC was measured using a thermal expansion analyzer (DIL 402CL, Netzsch).

##### Cell Fabrication

SFNbx|BZCYYb|SFNbx symmetrical cells were prepared to study the electrochemical performance of SFNbx as an air electrode. First, BZCYYb electrolyte disks and SFNbx electrode slurry were prepared. The SFNbx electrode slurry was prepared by mixing SFNbx powder with appropriate amounts of isopropanol (AR, 99.5%), ethylene glycol (AR, 99.5%), and glycerol (AR, 99.5%) until homogeneous.

BZCYYb electrolyte powder, prepared via the sol‐gel method, was pressed into green bodies using a mold and then sintered at 1450 °C for 5 h to achieve densification. The SFNbx electrode slurry was uniformly sprayed onto the electrolyte surface and sintered at 1000 °C for 2 h to obtain the desired symmetrical cell. A layer of silver paste was applied to the electrode surface as a current collector.

Ni–BZCYYb|BZCYYb|SFNbx single cells were prepared using a co‐pressing method. First, NiO powder (FuelCellMaterials Co. Ltd), BZCYY powder, and cornstarch were mixed uniformly in a mass ratio of 6.5:3.5:1 to obtain the fuel electrode powder, with cornstarch serving as the pore former. Next, 0.35 g of fuel electrode powder was pressed into disks using a mold. Then, 0.015 g of BZCYYb was uniformly spread on the fuel electrode disk and co‐pressed to obtain a NiO–BZCYYb half‐cell. The half‐cell was then sintered at 1450 °C for 5 h in a high‐temperature furnace. The SFNbx air electrode slurry was uniformly sprayed onto the exposed electrolyte side and sintered at 1000 °C for 2 h to obtain the desired single cell.

##### Electrochemical Performance Characterization

The electrochemical performance of the SFNbx air electrode was characterized through symmetrical cell performance testing. An electrochemical workstation (Solartron 2480) recorded the EIS of the cell under different atmospheres and temperatures. The testing atmospheres included dry air, 3 vol% H_2_O–air, and 6 vol% H_2_O–air, with a controlled gas flow rate of 50 mL min^−1^. The temperature testing sequence ranged from 700 to 450 °C, with measurements taken at 50 °C intervals. During testing, a 30 min stabilization period was implemented after each change in atmospheric conditions or temperature before conducting measurements.

The polarization resistance of the cell was analyzed using the DRT tool to obtain kinetic information on the elementary reactions of the air electrode. The frequency range for EIS testing was 10^5^–10^−1^ Hz, with an amplitude of 20 mV.

Additionally, the practical application of SFNbx as a RePCC air electrode was evaluated through single‐cell performance testing. Homemade molds were used for testing. The single cell was mounted on a ceramic mold, with silver wire leads connected to the fuel electrode and air electrode. Cell voltage and current measurements were conducted using a four‐probe method with a digital source meter (Keithley 2440). The humidified testing atmosphere was generated using a water bath‐heated bubbler system, with precise control of vapor content achieved through water bath temperature regulation. The temperature‐controlled furnace (OTF‐1200X‐S‐VT) was supplied by Hefei Kejing Materials Technology Co., Ltd.

Prior to experimental testing, comprehensive calibration and verification procedures were performed on both the electrochemical workstation and digital source meter instruments. The calibration protocol consisted of: 1) connecting multiple reference resistors with precisely known impedance values to each instrument, 2) applying identical testing parameters to those used in actual cell measurements, 3) conducting multiple measurements for each reference resistor, 4) recording and analyzing the acquired data to determine corresponding resistance values, and 5) comparing these measured values against certified standard impedance references to validate instrument accuracy.

## Conflict of Interest

The authors declare no conflict of interest.

## Supporting information

Supplementary Material

## Data Availability

The data that support the findings of this study are available from the corresponding author upon reasonable request.
