# Supplementary material for: Engineering Synergistic Oxygen‐Proton Properties for High‐Performance Reversible Protonic Ceramic Cell Air Electrodes
Source: Small Sci. 2025 Jul 22;5(10):2500256. doi: 10.1002/smsc.202500256 (PMC12499451; doi:10.1002/smsc.202500256)
Supplement: Supplementary file 1 — Supplementary Material [file SMSC-5-2500256-s001.pdf]

Supporting Information

Engineering Synergistic Oxygen-Proton Properties for High-Performance Reversible Protonic Ceramic Cell Air Electrodes

Na Yu[a], Xi Chen[a], Tong Liu[a,b], Shuo Zhai[a,c], Jiaxin Yuan[a], Yufei Song[d], Meng Ni[a]**

1. Supporting Figures

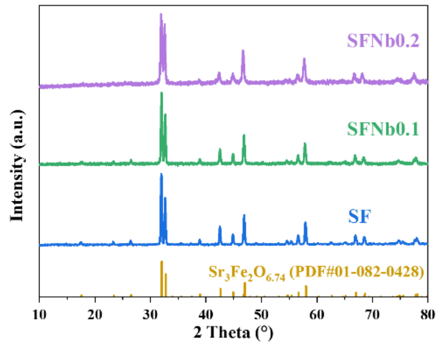

Figure S1 XRD patterns of fresh SFNbx powders.

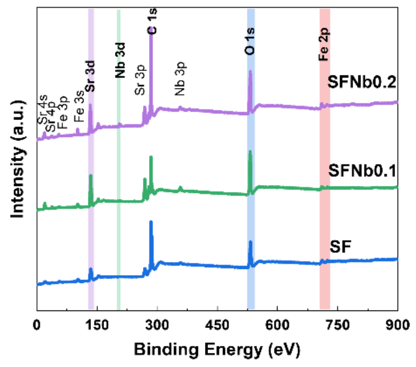

Figure S2 XPS sum spectra of SFNbx materials.

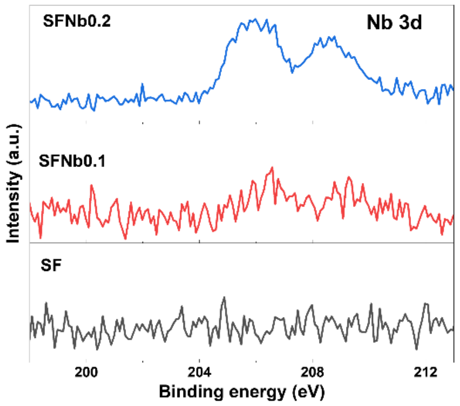

Figure S3 XPS spectra of SFNbx materials: Nb 3d.

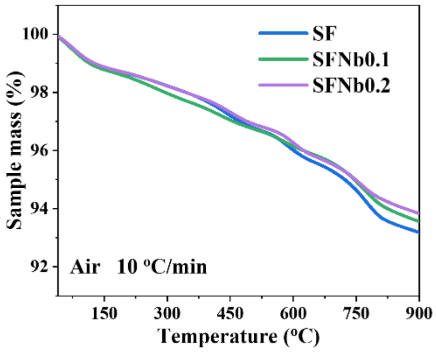

Figure S4 TG curves of the as-prepared SFNbx samples in the temperature range from room temperature to 900 °C.

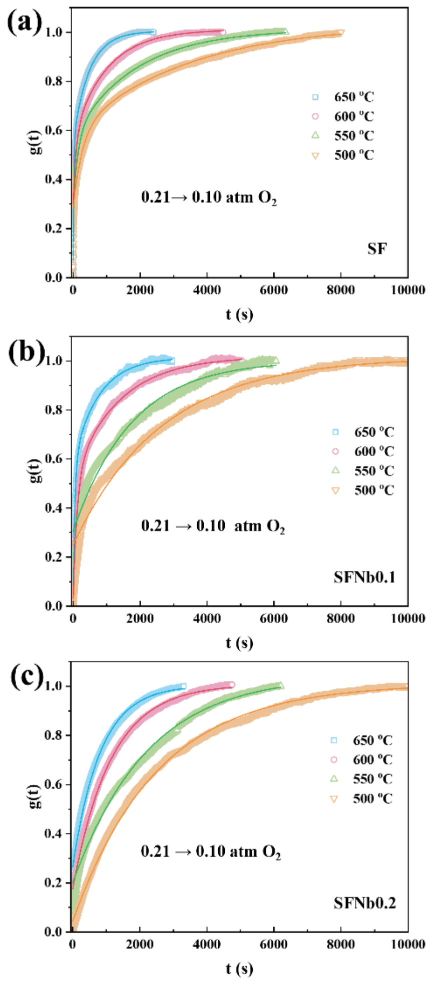

Figure S5 ECR curves measured during the transition from 21% O_2–79% N_2 to 10% O_2–90% N_2 atmospheres.

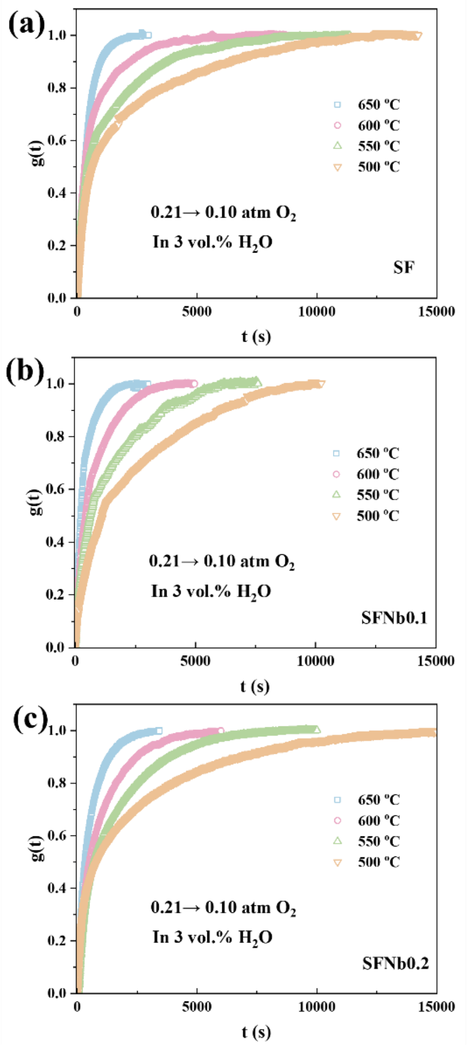

Figure S6 ECR curves of SFNbx samples during the transition from 21% O_2–76% N_2–3% H_2O to 10% O_2–87% N_2–3% H_2O atmospheres.

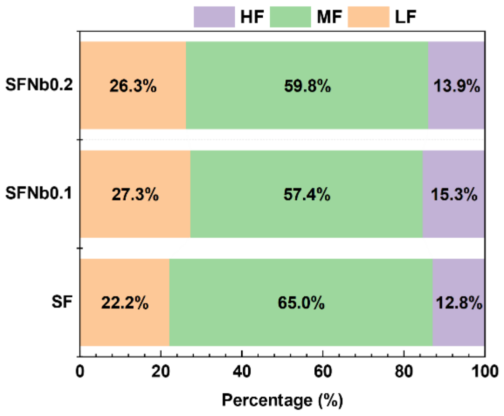

Figure S7 The proportions of the resolved peaks calculated from the DRT results shown in Figure 3d.

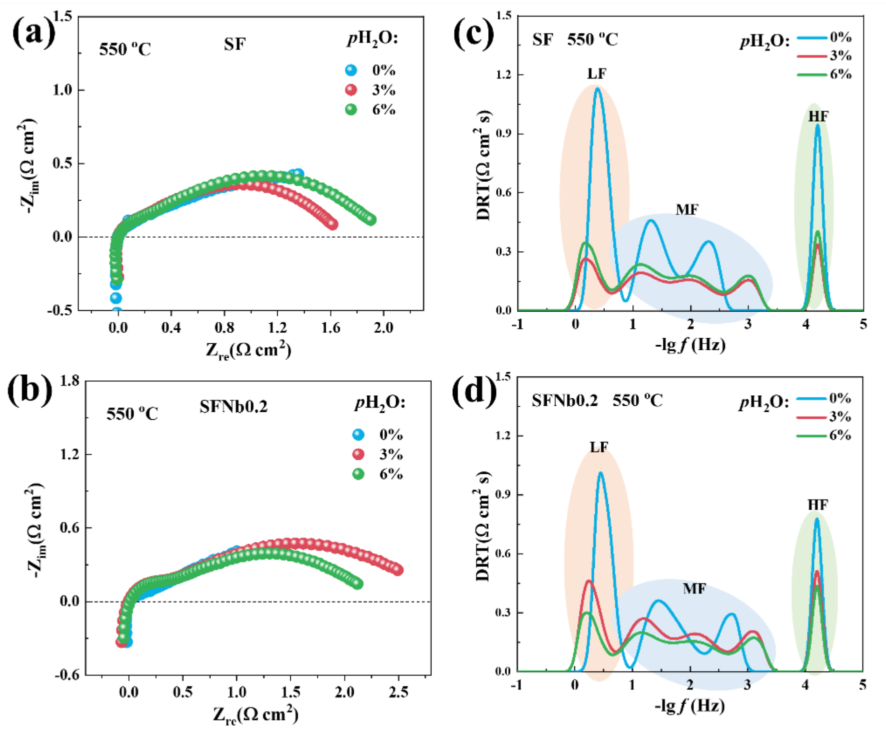

Figure S8 Effect of water pressure on the performance of SF and SFNb0.2 electrodes. (a-b) EIS spectra under different water pressures. (c-d) Corresponding DRT analysis results.

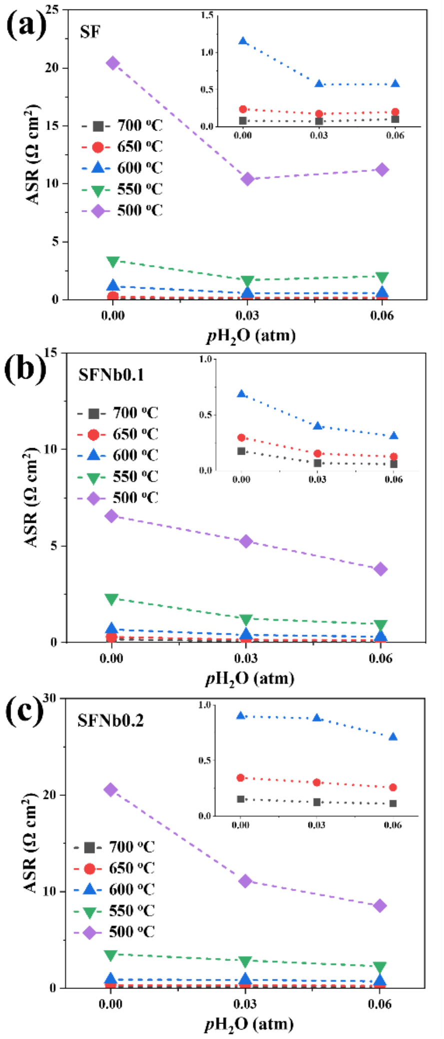

Figure S9 Polarization resistance of SFNbx electrodes in the temperature range of 500-700 °C as a function of water partial pressure

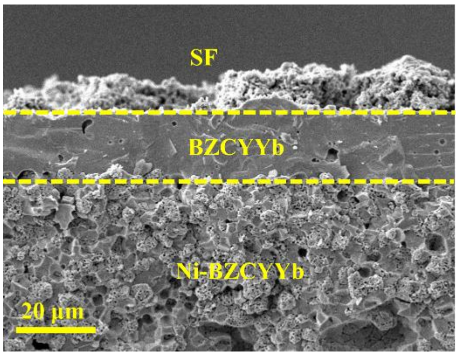

Figure S10 Cross-sectional SEM image of the NiO-BZCYYb/BZCYYb/SF single cell after electrochemical testing.

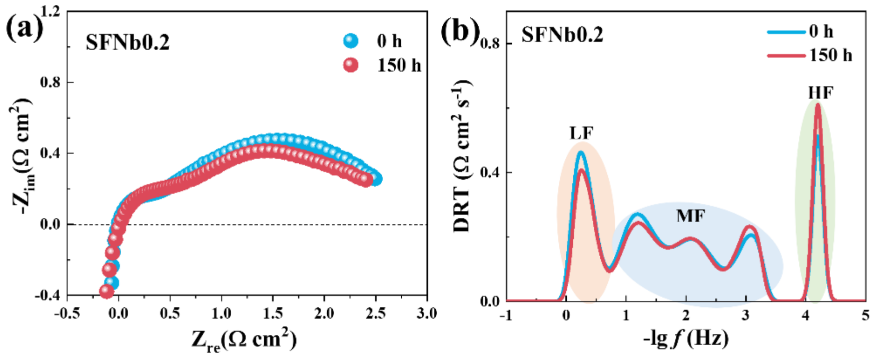

Figure S11 (a) EIS and (b) DRT curves before and after long-term testing for SFNb0.2 electrode.

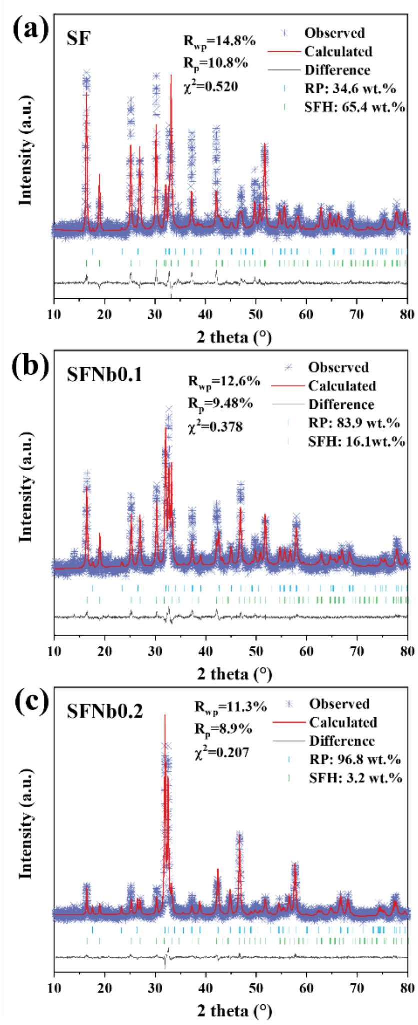

Figure S12 XRD refinement results of SFNbx samples treated in wet air for 150 h.

2. Supporting Tables

Table S1 Results of the refined XRD patterns.

Lattice parameter	SF	SFNb0.1	SFNb0.2
a (Å)	3.86587	3.87972	3.89263
b (Å)	3.86587	3.87972	3.89263
c (Å)	20.15923	20.20369	20.21467
Space group	I4/mmm	I4/mmm	I4/mmm
Volume (nm^3)	0.30128	0.30411	0.30630
Crystallite size (nm)	33.02	29.29	23.74

Table S2 The performance comparison of SFNbx air electrodes with that of advanced air electrodes reported in the literature under conditions of humidified air at 450-700 °C.

Air Electrode	Electrolyte	ASR (Ω cm^2)						Ref.
		700 °C	650 °C	600 °C	550 °C	500 °C	450 °C	
SF	BZCYYb	0.07	0.17	0.57	1.69	10.41	60.44	This work
SFNb0.1	BZCYYb	0.07	0.15	0.40	1.25	5.25	22.81	This work
SFNb0.2	BZCYYb	0.13	0.30	0.88	2.88	11.11	43.04	This work
PNO	BCY91	0.59		1.50	6.78	24.29	78.35	[1]
CCO	BCY91	0.41	0.89	2.20	6.33	14.87	53.31	[2]
CLCO	BCY91	0.36	0.78	2.20	3.85	9.01	28.93	[2]
BCFZ	BZCYYb		0.60	1.02	1.90	3.89	8.43	[3]
LSC	BZCY	0.55	1.7	3.25	4.0	5.0		[4]
BCFZY	BZCYYb	0.89	1.72	4.01	7.69	16.12		[5]
PBCC-C	BZCYYb	0.24	0.68	1.72	5.30			[6]
BSCFT10	BZCY442	0.89	1.56	3.12	4.39			[7]
BSCF	BZCY442	0.79	1.33	1.96	3.99			[7]
3-$SEFC_{0.5}$	BZCY352	0.37	0.95	2.78				[8]
2W-PBSCF	BZCYYb		0.24	0.36	0.80	1.39		[9]
PBSCF	BZCYYb	0.29	0.77	1.97	4.18	11.70		[10]
16F-PBSCF	BZCYYb	0.10	0.28	0.49	1.21	3.06		[10]
HE-PBSLCC	BZCYYb	0.08	0.15	0.35	0.95	2.5		[11]
NBCFM	BZCYYb	0.28	0.50	0.99	2.19	5.17		[12]
LSCF	BCY20	0.06	0.40	0.79				[13]
SCFN	BZCYYb		0.08	0.20	0.57	2.05	8.46	[14]

Note: PNO: Pr_2NiO_4; CCO: $Ca_3Co_4O_9$; CLCO: $Ca_{2.7}La_{0.3}Co_4O_9$; LSC: $La_{0.6}Sr_{0.4}CoO_{3-\delta}$; BCFZ:

$BaCo_{0.4}Fe_{0.4}Zr_{0.2}O_{3-\delta}$; BCFZY: $BaCo_{0.4}Fe_{0.4}Zr_{0.1}Y_{0.1}O_{3-\delta}$; PBCC-C: $PrBa_{0.8}Ca_{0.2}Co_2O_{5+\delta}$; BSCFT10: $Ba_{0.5}Sr_{0.5}Co_{0.72}Fe_{0.18}Ti_{0.10}O_{3-\delta}$; BSCF: $Ba_{0.5}Sr_{0.5}Co_{0.8}Fe_{0.2}O_{3-\delta}$; 3-SEFC0.5: $Sr_3EuFe_{2.5}Co_{0.5}O_{10-\delta}$; PBSCF: $PrBa_{0.5}Sr_{0.5}Co_{1.5}Fe_{0.5}O_{5+\delta}$; 2W-PBSCF: 2 wt.% $(NH_4)_{10}W_{12}O_{41}\cdot 5H_2O$–PBSCF; 16F-PBSCF: $PrBa_{0.5}Sr_{0.5}Co_{1.5}Fe_{0.5}O_{5.84-\delta}F_{0.16}$; HE-PBSLCC: $Pr_{0.2}Ba_{0.2}Sr_{0.2}La_{0.2}Ca_{0.2}CoO_{3-\delta}$; NBCFM: $Nd_{1.5}Ba_{1.5}CoFeMnO_{9-\delta}$; LSCF: $La_{0.6}Sr_{0.4}Co_{0.2}Fe_{0.8}O_{3-\delta}$; SCFN: $Sr_{0.9}Ce_{0.1}Fe_{0.8}Ni_{0.2}O_{3-\delta}$; BZCYYb: $BaZr_{0.1}Ce_{0.7}Y_{0.1}Yb_{0.1}O_{3-\delta}$; BCY91: $BaCe_{0.9}Y_{0.1}O_{3-\delta}$; BZCY: $BaCe_{0.54}Zr_{0.36}Y_{0.1}O_{2.95}$; BZCY442: $BaZr_{0.4}Ce_{0.4}Y_{0.2}O_{3-\delta}$; BZCY352: $BaZr_{0.3}Ce_{0.5}Y_{0.2}O_{3-\delta}$; BCY20: $BaCe_{0.8}Y_{0.2}O_{3-\delta}$;

Table S3 Comparison of the performance of the single cell with SFNb0.1 electrode to previously reported single cell performances

Air electrode	Electrolyte (μm)	Fuel/Oxidant	Peak power density (mW cm^{-2})						Ref.
			700 ℃	650 ℃	600 ℃	550 ℃	500 ℃	450 ℃	
SF	BZCYYb (17 μm)	H_2/3%H_2O-air		468	319	214	144	85	This work
SFNb0.1	BZCYYb (17 μm)	H_2/3%H_2O-air		576	442	302	188	105	This work
SSC	BZCYYb (11 μm)	3%H_2O-H_2/Air		611	332	137	56		[15]
STF	BZCYYb (19 μm)	H_2/3%H_2O-air			471	321	190		[16]
K10	BZCYYb4411 (20 μm)	3%H_2O-H_2/3%H_2O-Air	836	667	502				[17]
BCFZ442	BZCYYb4411 (20 μm)	3%H_2O-H_2/3%H_2O-Air	684	560	421				[17]
16F-PBSCF	BZCYYb (8 μm)	3%H_2O-H_2/Air		580	510	300			[10]
SCF	BZCYYb (10 μm)	H_2/Air		571	417	296	203		[18]
SCFP	BZCYYb (10 μm)	H_2/Air		865	601	372	278		[18]
PNC	BZCY (5 μm)	3%H_2O-H_2/Air	416	284	171				[19]
PNC/B	BZCY (5	3%H_2O-	558	391	218				[19]

ZCY	μm)	H_2/Air							
BCFZW	BZCYYb (20 μm)	3%H_2O-H_2/Air		762	686	593	493	394	[20]
LSF	BCZY (15 μm)	H_2/Air	606	466	307				[21]
BSCFW	BZCYYb (15 μm)	3%H_2O-H_2/Air		873	582	421	126		[22]
BZFY-6	BZCYYb (25 μm)	3%H_2O-H_2/Air	322	217	172	109			[23]
BCFB	BZCY (25 μm)	H_2/3%-Air	736	551	362	203			[24]
BCFC	BZCYYb (70 μm)	H_2/3%-Air	335	287	237				[25]
BFB	BZCY (14 μm)	H_2/3%-Air	635	479	338	282			[26]
PBCCY02	BZCYYb (11μm)	3%H_2O-H_2/3H_2O-Air		803	516	354			[27]
BLFZ	BZCYYb (15μm)	3%H_2O-H_2/Air	630	510	370				[28]
BZFY07	BZCYYb (13μm)	3%H_2O-H_2/Air	620	550	380	210			[29]

Note:

SSC: $Sm_{0.5}Sr_{0.5}CoO_{3-\delta}$; STF: $SrTi_{0.1}Fe_{0.9}O_{3-\delta}$; K10: $Ba_{0.9}K_{0.1}Co_{0.4}Fe_{0.4}Zr_{0.2}O_{3-\delta}$; BCFZ442: $BaCo_{0.4}Fe_{0.4}Zr_{0.2}O_{3-\delta}$; 16F-PBSCF: $PrBa_{0.5}Sr_{0.5}Co_{1.5}Fe_{0.5}O_{5.84-\delta}F_{0.16}$; SCF: $SrCo_{0.8}Fe_{0.2}O_{3-\delta}$; SCFP: $SrCo_{0.8}Fe_{0.15}P_{0.05}O_{3-\delta}$; PNC: $PrNi_{0.5}Co_{0.5}O_{3-\delta}$; PNC/BZCY: PNC/$BaCe_{0.7}Zr_{0.1}Y_{0.2}O_{3-\delta}$; BCFZW: $BaCo_{0.4}Fe_{0.4}Zr_{0.15}W_{0.05}O_{3-\delta}$; LSF: $La_{0.5}Sr_{0.5}FeO_{3-\delta}$; BSCFW: $Ba_{0.5}Sr_{0.5}(Co_{0.7}Fe_{0.3})_{0.6875}W_{0.3125}O_{3-\delta}$; BZFY-6: $BaZr_{0.2}Fe_{0.6}Y_{0.2}O_{3-\delta}$; BCFB: $BaCe_{0.5}Fe_{0.3}Bi_{0.2}O_{3-\delta}$; BCFC: $BaCe_{0.4}Fe_{0.4}Co_{0.2}O_{3-\delta}$; BFB: $BaFe_{0.9}Bi_{0.1}O_{3-\delta}$; BZCYYb: $BaZr_{0.1}Ce_{0.7}Y_{0.1}Yb_{0.1}O_{3-\delta}$; BZCYYb4411: $BaZr_{0.4}Ce_{0.4}Y_{0.1}Yb_{0.1}O_{3-\delta}$; BZCY: $BaZr_{0.1}Ce_{0.7}Y_{0.2}O_{3-\delta}$; PBCCY02: $Pr_{0.2}Ba_{0.8}Co_{0.4}Ce_{0.4}Y_{0.2}O_{3-\delta}$; BLFZ: $Ba_{0.95}La_{0.05}Fe_{0.8}Zn_{0.2}O_{3-\delta}$; BZFY07: $BaZr_{0.18}Fe_{0.7}Y_{0.12}O_{3-\delta}$.

Table S4 Comparison of the performance of the PCEC with SFNb0.1 electrode to previously reported cell performances under same operating condition.

Air electrode	Electrolyte (μm)	Fuel/Oxidant	Current density (Acm^{-2}) at 1.3V						Ref.
			700 ℃	650 ℃	600 ℃	550 ℃	500 ℃	450 ℃	

SF	BZCYYb (17 μm)	H_2/3%H_2O-air		-0.84	-0.55	-0.32	-0.21	-0.11	This work
SFNb0.1	BZCYYb (17 μm)	H_2/3%H_2O-air		-1.14	-0.86	-0.52	-0.29	-0.19	This work
BCCF	BZCYYb (10 μm)	H_2/3%H_2O-air		-2.25	-1.62	-0.96			[30]
PBCC	BZCYYb (10 μm)	3%H_2O-H_2/3%H_2O-air		-2.52	-1.51	-0.69			[31]
D-SFN	BZCYYb (17 μm)	H_2/3%H_2O-air		-1.19	-0.81	-0.57	-0.29	-0.17	[32]
BSCF	BZCYYb (16.4 μm)	H_2/3%H_2O-air		-0.41	-0.33	-0.21	-0.11		[33]
BSCFF	BZCYYb (16.6 μm)	H_2/3%H_2O-air		-0.95	-0.59	-0.316	-0.19		[33]
BSCFCl	BZCYYb (17.6 μm)	H_2/3%H_2O-air		-0.67	-0.49	-0.29	-0.14		[33]
SCFN	BZCYYb (26 μm)	H_2/3%H_2O-air			-0.36	-0.27	-0.18	-0.09	[14]
SEFC-BZCY	BZCY (15 μm)	3%H_2O-H_2/10%H_2O-air	-1.03	-0.66	-0.38				[34]
BCFZY	BZCYYb (15 μm)	H_2/10% H_2O-air			-0.84	-0.58	-0.38	-0.22	[35]
PNC	BZCYYb (10 μm)	H_2 /10% H_2O-air			-0.86	-0.48	-0.34	-0.12	[36]
SLF	BZCY (20 μm)	3%H_2O-H_2/20% H_2O-air	-1.07	-0.72	-0.46				[37]
NBN	BCZD (15 μm)	3%H_2O-H_2/3% H_2O-ai	-040	-0.28	-0.16	-0.09	-0.05		[38]
BSCFP0.05	BZCYYb (10 μm)	H_2/3% H_2O-air			-1.00	-0.62	-0.4		[39]
LN-BCZD	BCZD (30 μm)	3%H_2O-H_2/3% H_2O-air	-0.30		-0.16				[40]
BLFZ	BZCYYb (15 μm)	3%H_2O-H_2/10%H_2O-Air			-0.41				[28]

BZFY07	BZCYYb (13 μm)	3%H_2O-H_2/3%H_2O-Air	-0.95		-0.45	[27]
BLFZN0.1	BZCYYb (~13 μm)	3%H_2O-H_2/3%H_2O-Air	-1.66	-1.09	-0.67	[41]

Note:

BCCF: $Ba_{0.95}Cs_{0.05}Ce_{0.3}Fe_{0.7}O_{3+\delta}$; PBCC: $PrBa_{0.8}Ca_{0.2}Co_2O_{5+\delta}$; D-SFN: $Sr_{2.8}Fe_{1.8}Nb_{0.2}O_{7-\delta}$; BSCF: $Ba_{0.5}Sr_{0.5}Co_{0.8}Fe_{0.2}O_{3-\delta}$; BSCFF: $Ba_{0.5}Sr_{0.5}Co_{0.8}Fe_{0.2}O_{2.9-\sigma}F_{0.1}$; BSCFCl: $Ba_{0.5}Sr_{0.5}Co_{0.8}Fe_{0.2}O_{2.9-\sigma}Cl_{0.1}$; SCFN: $Sr_{0.9}Ce_{0.1}Fe_{0.8}Ni_{0.2}O_{3-\delta}$; SEFC-BZCY: $SrEu_2Fe_{1.8}Co_{0.2}O_{7-\delta}$-5wt.%BZCY; BCFZY: $BaCo_{0.4}Fe_{0.4}Zr_{0.1}Y_{0.1}O_{3-\delta}$; PNC: $PrNi_{0.5}Co_{0.5}O_{3-\delta}$; SLF: $Sr_{2.8}La_{0.2}Fe_2O_{7-\delta}$; NBN: $Nd_{1.95}Ba_{0.05}NiO_4$; BSCFP0.05: $Ba_{0.5}Sr_{0.5}(Co_{0.8}Fe_{0.2})_{0.95}P_{0.05}O_{3-\delta}$; LN-BCZD: $La_2NiO_{4+\delta}$-$BaCe_{0.5}Zr_{0.3}Dy_{0.2}O_{3-\delta}$; BCZD: $BaCe_{0.5}Zr_{0.3}Dy_{0.2}O_{3-\delta}$; BLFZ: $Ba_{0.95}La_{0.05}Fe_{0.8}Zn_{0.2}O_{3-\delta}$; BZFY07: $BaZr_{0.18}Fe_{0.7}Y_{0.12}O_{3-\delta}$; BLFZN0.1: $Ba_{0.95}La_{0.05}(Fe_{0.8}Zn_{0.2})_{0.9}Ni_{0.1}O_{3-\delta}$.

Table S5 Comparison of TEC of SFNbx with common electrode materials reported in the literature

Electrode material	TEC ($\cdot 10^{-6}$ K^{-1})	Ref.
BCN	24.2	[42]
BC1.5MN	21.1	[42]
BCM	22.0	[42]
LSCSb	22.5	[43]
GSC	23.0	[44]
NBCO	20.9	[45]
NBCO-8CGO	20.1	[45]
LBSC	26.2	[46]
NBSC	23.3	[47]
SF	19.6	This work
SFNb0.1	18.8	This work
SFNb0.2	17.7	This work

Note: NBSCF: $NdBa_{0.5}Sr_{0.5}Co_{1.5}Fe_{0.5}O_{5+\delta}$; SCT: $SrCo_{0.95}Ta_{0.05}O_{3-\delta}$; SCT); BCN: $BaCoNbO_{3-\delta}$; BC1.5MN: $Ba_2Co_{1.5}Mo_{0.25}Nb_{0.25}O_{6-\delta}$; BCM: $BaCoMoO_{3-\delta}$; LSCSb: $La_{0.4}Sr_{0.6}Co_{0.9}Sb_{0.1}O_{3-\delta}$; GSC: $Gd_{0.8}Sr_{0.2}CoO_{3-\delta}$; NBCO: $NdBaCo_2O_{5+\delta}$; NBCO-8CGO: NBCO-$Ce_{0.9}Gd_{0.1}O_{1.95}$. LBSC: $LaBa_{0.5}Sr_{0.5}Co_2O_{5+\delta}$; NBSC: $NdBa_{0.5}Sr_{0.5}Co_2O_{5+\delta}$.

Supporting References

1. Dailly, J.; Fourcade, S.; Largeteau, A.; Mauvy, F.; Grenier, J. C.; Marrony, M., Perovskite and A_2MO_4-type oxides as new cathode materials for protonic solid oxide fuel

cells, *Electrochim. Acta* **2010,** *55* (20), 5847-5853, https://doi.org/10.1016/j.electacta.2010.05.034.
2. Yahia, H. B.; Mauvy, F.; Grenier, J. C., $Ca_{3-x}La_xCo_4O_{9+\delta}$ (x=0, 0.3): New cobaltite materials as cathodes for proton conducting solid oxide fuel cell, *J. Solid State Chem.* **2010,** *183* (3), 527-531, https://doi.org/10.1016/j.jssc.2009.12.014.
3. Shang, M.; Tong, J.; O'Hayre, R., A promising cathode for intermediate temperature protonic ceramic fuel cells: $BaCo_{0.4}Fe_{0.4}Zr_{0.2}O_{3-\delta}$, *Rsc. Adv.* **2013,** *3* (36), 15769-15775, https://doi.org/10.1039/C3RA41828F.
4. Samat, A. A.; Jais, A. A.; Somalu, M. R.; Osman, N.; Muchtar, A.; Lim, K. L., Electrical and electrochemical characteristics of $La_{0.6}Sr_{0.4}CoO_{3-\delta}$ cathode materials synthesized by a modified citrate-EDTA sol-gel method assisted with activated carbon for proton-conducting solid oxide fuel cell application, *J. Sol-Gel Sci. Technol.* **2018,** *86* (3), 617-630, https://doi.org/10.1007/s10971-018-4675-1.
5. Duan, C.; Tong, J.; Shang, M.; Nikodemski, S.; Sanders, M.; Ricote, S.; Almansoori, A.; O'Hayre, R., Readily processed protonic ceramic fuel cells with high performance at low temperatures, *Science* **2015,** *349* (6254), 1321-1326, https://doi.org/10.1126/science.aab3987.
6. Zhang, H.; Xu, K.; Xu, Y.; He, F.; Zhu, F.; Zhu, L.; Chen, Y., Improving the performance of the $PrBa_{0.8}Ca_{0.2}Co_2O_{5+\delta}$ cathode for proton-conducting SOFCs by microwave sintering, *Ceram. Int.* **2024,** *50* (20, Part C), 40384-40390, https://doi.org/10.1016/j.ceramint.2024.05.299.
7. Bi, L.; Fabbri, E.; Traversa, E., Novel $Ba_{0.5}Sr_{0.5}(Co_{0.8}Fe_{0.2})_{1-x}Ti_xO_{3-\delta}$ (x=0, 0.05, and 0.1) cathode materials for proton-conducting solid oxide fuel cells, *Solid State Ionics* **2012,** *214*, 1-5, https://doi.org/10.1016/j.ssi.2012.02.049.
8. Huan, D.; Zhang, L.; Li, X.; Xie, Y.; Shi, N.; Xue, S.; Xia, C.; Peng, R.; Lu, Y., A durable Ruddlesden-Popper cathode for protonic ceramic fuel cells, *ChemSusChem* **2020,** *13* (18), 4994-5003, https://doi.org/10.1002/cssc.202001168.
9. Zhao, S.; Ma, W.; Wang, W.; Huang, Y.; Wang, J.; Wang, S.; Shu, Z.; He, B.; Zhao, L., Reverse atom capture on perovskite surface enabling robust and efficient cathode for protonic ceramic fuel cells, *Adv. Mater.* **2024,** *36* (27), 2405052, https://doi.org/10.1002/adma.202405052.
10. Xu, Y.; Huang, Y.; Guo, Y.; Hu, F.; Xu, J.; Zhou, W.; Yang, Z.; Sun, J.; He, B.; Zhao, L., Engineering anion defect in perovskite oxyfluoride cathodes enables proton involved oxygen reduction reaction for protonic ceramic fuel cells, *Sep. Purif. Technol.* **2022,** *290*, 120844, https://doi.org/10.1016/j.seppur.2022.120844.
11. He, F.; Zhou, Y.; Hu, T.; Xu, Y.; Hou, M.; Zhu, F.; Liu, D.; Zhang, H.; Xu, K.; Liu, M.; Chen, Y., An efficient high-entropy perovskite-type air electrode for reversible oxygen reduction and water splitting in protonic ceramic cells, *Adv. Mater.* **2023,** *35* (16), 2209469, https://doi.org/10.1002/adma.202209469.
12. Lee, J.-I.; Park, K.-Y.; Park, H.; Bae, H.; Saqib, M.; Park, K.; Shin, J.-S.; Jo, M.; Kim, J.; Song, S.-J.; Wachsman, E. D.; Park, J.-Y., Triple perovskite structured $Nd_{1.5}Ba_{1.5}CoFeMnO_{9-\delta}$ oxygen electrode materials for highly efficient and stable reversible protonic ceramic cells, *J. Power Sources* **2021,** *510*, 230409, https://doi.org/10.1016/j.jpowsour.2021.230409.
13. Miyazaki, K.; Ding, Y.; Muroyama, H.; Matsui, T.; Eguchi, K., $La_{0.6}Sr_{0.4}Co_{0.2}Fe_{0.8}O_{3-\delta}$–Ba(Ce,Co,Y)$O_{3-\delta}$ composite cathodes for proton-conducting ceramic fuel cells, *Electrochemistry* **2020,** *88* (1), 28-33, https://doi.org/10.5796/electrochemistry.19-00039.
14. Song, Y.; Liu, J.; Wang, Y.; Guan, D.; Seong, A.; Liang, M.; Robson, M. J.; Xiong, X.; Zhang, Z.; Kim, G.; Shao, Z.; Ciucci, F., Nanocomposites: A new opportunity for developing highly active and durable bifunctional air electrodes for reversible protonic ceramic cells, *Adv. Energy Mater.* **2021,** *11* (36), 2101899, https://doi.org/10.1002/aenm.202101899.
15. Seong, A.; Jeong, D.; Kim, M.; Choi, S.; Kim, G., Performance comparison of composite cathode: Mixed ionic and electronic conductor and triple ionic and electronic conductor with

$BaZr_{0.1}Ce_{0.7}Y_{0.1}Yb_{0.1}O_{3-\delta}$ for highly efficient protonic ceramic fuel cells, *J. Power Sources* **2022,** *530*, 231241, https://doi.org/10.1016/j.jpowsour.2022.231241.
16. Zhou, C.; Liu, D.; Fei, M.; Wang, X.; Ran, R.; Xu, M.; Wang, W.; Zhou, W.; O'Hayre, R.; Shao, Z., Cathode water management towards improved performance of protonic ceramic fuel cells, *J. Power Sources* **2023,** *556*, 232403, https://doi.org/10.1016/j.jpowsour.2022.232403.
17. Qiu, P.; Liu, B.; Wu, L.; Qi, H.; Tu, B.; Li, J.; Jia, L., K-doped $BaCo_{0.4}Fe_{0.4}Zr_{0.2}O_{3-\delta}$ as a promising cathode material for protonic ceramic fuel cells, *J. Adv. Ceram.* **2022,** *11* (12), 1988-2000, https://doi.org/10.1007/s40145-022-0662-7.
18. Liu, Z.; Hu, Z.; Di, H.; Yang, M.; Yang, G.; Wang, W.; Ran, R.; Zhou, W., High-performance phosphorus-doped $SrCo_{0.8}Fe_{0.2}O_{3-\delta}$ cathode for protonic ceramic fuel cells, *Ceram. Int.* **2024,** *50* (20, Part C), 40409-40416, https://doi.org/10.1016/j.ceramint.2024.03.087.
19. Yuan, C.; Tong, X.; Li, C.; Sun, Z.; Li, P.; Zhang, Y.; Wang, N.; Shen, X.; Zhan, Z.; Wang, L., Dual Self-Assembled Nanocomposite Cathode for Protonic Ceramic Fuel Cells, *Energy & Fuels* **2024,** *38* (3), 2396-2403, https://doi.org/10.1021/acs.energyfuels.3c04882.
20. Yang, J.; Zhou, C.; Zheng, S.; Zhang, L., Accelerated oxygen reduction kinetics in $BaCo_{0.4}Fe_{0.4}Zr_{0.2}O_{3-\delta}$ cathode via doping with a trace amount of tungsten for protonic ceramic fuel cells, *Ceram. Int.* **2024,** *50* (22, Part A), 44935-44942, https://doi.org/10.1016/j.ceramint.2024.08.331.
21. He, S.; Dai, H.; Bi, L., A highly efficient Sb-doped $La_{0.5}Sr_{0.5}FeO_{3-\delta}$ cathode for protonic ceramic fuel cells, *Ceram. Int.* **2024,** *50* (1, Part A), 1284-1292, https://doi.org/10.1016/j.ceramint.2023.10.090.
22. Hu, D.; Kim, J.; Niu, H.; Daniels, L. M.; Manning, T. D.; Chen, R.; Liu, B.; Feetham, R.; Claridge, J. B.; Rosseinsky, M. J., High-performance protonic ceramic fuel cell cathode using protophilic mixed ion and electron conducting material, *J. Mater. Chem. A* **2022,** *10* (5), 2559-2566, https://doi.org/10.1039/D1TA07113K.
23. Wu, Y.; Li, K.; Yang, Y.; Song, W.; Ma, Z.; Chen, H.; Ou, X.; Zhao, L.; Khan, M.; Ling, Y., Investigation of Fe-substituted in $BaZr_{0.8}Y_{0.2}O_{3-\delta}$ proton conducting oxides as cathode materials for protonic ceramics fuel cells, *J. Alloys Compd.* **2020,** *814*, 152220, https://doi.org/10.1016/j.jallcom.2019.152220.
24. Shan, D.; Gong, Z.; Wu, Y.; Miao, L.; Dong, K.; Liu, W., A novel $BaCe_{0.5}Fe_{0.3}Bi_{0.2}O_{3-\delta}$ perovskite-type cathode for proton-conducting solid oxide fuel cells, *Ceram. Int.* **2017,** *43* (4), 3660-3663, https://doi.org/10.1016/j.ceramint.2016.11.206.
25. Zhao, Z.; Cui, J.; Zou, M.; Mu, S.; Huang, H.; Meng, Y.; He, K.; Brinkman, K. S.; Tong, J., Novel twin-perovskite nanocomposite of Ba-Ce-Fe-Co-O as a promising triple conducting cathode material for protonic ceramic fuel cells, *J. Power Sources* **2020,** *450*, 227609, https://doi.org/10.1016/j.jpowsour.2019.227609.
26. Xia, Y.; Xu, X.; Teng, Y.; Lv, H.; Jin, Z.; Wang, D.; Peng, R.; Liu, W., A novel $BaFe_{0.8}Zn_{0.1}Bi_{0.1}O_{3-\delta}$ cathode for proton conducting solid oxide fuel cells, *Ceram. Int.* **2020,** *46* (16, Part A), 25453-25459, https://doi.org/10.1016/j.ceramint.2020.07.015.
27. Wu, X.; Wang, J.; Tian, H.; Li, W.; Li, C.-X., An efficient and robust triple-phase nanocomposite air electrode for reversible proton ceramic fuel cells, *Chem. Eng. J.* **2025,** *512*, 162395, https://doi.org/10.1016/j.cej.2025.162395.
28. Bao, Y.; Tang, W.; Chen, P.; Lu, Y.; Hong, T.; Cheng, J., The evaluation of triple conductor Ba0.95La0.05Fe0.8Zn0.2O3-δ as air electrode for reversible protonic ceramic fuel cell, *J. Power Sources* **2025,** *628*, 235882, https://doi.org/10.1016/j.jpowsour.2024.235882.
29. Tang, W.; Li, C.; Xia, Q.; Lu, Y.-W.; Chen, P.; Hong, T.; Cheng, J., Tuning the proton concentration and uptake kinetics of $BaFeO_3$ based oxygen electrode for reversible protonic ceramic fuel cells, *J. Mater. Chem. A* **2025**, https://doi.org/10.1039/D5TA00459D.

30. Gao, Y.; Liu, K.; Zhang, X.; Li, Q.; Chang, Y.; Fu, M.; Tao, Z., Cs-Doped BCCF perovskite with enhanced surface proton acid sites for high-performance R-PCECs, *Chem. Eng. J.* **2025,** *504*, 158984, https://doi.org/10.1016/j.cej.2024.158984.
31. Zhou, Y.; Liu, E.; Chen, Y.; Liu, Y.; Zhang, L.; Zhang, W.; Luo, Z.; Kane, N.; Zhao, B.; Soule, L.; Niu, Y.; Ding, Y.; Ding, H.; Ding, D.; Liu, M., An active and robust air electrode for reversible protonic ceramic electrochemical cells, *ACS Energy Lett.* **2021,** *6* (4), 1511-1520, https://doi.org/10.1021/acsenergylett.1c00432.
32. Yu, N.; Bello, I. T.; Chen, X.; Liu, T.; Li, Z.; Song, Y.; Ni, M., Rational design of Ruddlesden–Popper perovskite ferrites as air electrode for highly active and durable reversible protonic ceramic cells, *Nano-micro Lett.* **2024,** *16* (1), 177, https://doi.org/10.1007/s40820-024-01397-2.
33. Chen, X.; Yu, N.; Bello, I. T.; Guan, D.; Li, Z.; Liu, T.; Liu, T.; Shao, Z.; Ni, M., Facile anion engineering: A pathway to realizing enhanced triple conductivity in oxygen electrodes for reversible protonic ceramic electrochemical cells, *Energy Stor. Mater.* **2023,** *63*, 103056, https://doi.org/10.1016/j.ensm.2023.103056.
34. Huan, D.; Shi, N.; Zhang, L.; Tan, W.; Xie, Y.; Wang, W.; Xia, C.; Peng, R.; Lu, Y., New, efficient, and reliable air electrode material for proton-conducting reversible solid oxide cells, *ACS Appl. Mater. Interfaces* **2018,** *10* (2), 1761-1770, https://doi.org/10.1021/acsami.7b16703.
35. Liang, M.; Song, Y.; Liu, D.; Xu, L.; Xu, M.; Yang, G.; Wang, W.; Zhou, W.; Ran, R.; Shao, Z., Magnesium tuned triple conductivity and bifunctionality of $BaCo_{0.4}Fe_{0.4}Zr_{0.1}Y_{0.1}O_{3-\delta}$ perovskite towards reversible protonic ceramic electrochemical cells, *Appl. Catal. B-Environ.* **2022,** *318*, 121868, https://doi.org/10.1016/j.apcatb.2022.121868.
36. Ding, H.; Wu, W.; Jiang, C.; Ding, Y.; Bian, W.; Hu, B.; Singh, P.; Orme, C. J.; Wang, L.; Zhang, Y., Self-sustainable protonic ceramic electrochemical cells using a triple conducting electrode for hydrogen and power production, *Nat. Commun.* **2020,** *11* (1), 1907, https://doi.org/10.1038/s41467-020-15677-z.
37. Huan, D.; Wang, W.; Xie, Y.; Shi, N.; Wan, Y.; Xia, C.; Peng, R.; Lu, Y., Investigation of real polarization resistance for electrode performance in proton-conducting electrolysis cells, *J. Mater. Chem. A* **2018,** *6* (38), 18508-18517, https://doi.org/10.1039/C8TA06862C.
38. Danilov, N.; Lyagaeva, J.; Vdovin, G.; Pikalova, E.; Medvedev, D., Electricity/hydrogen conversion by the means of a protonic ceramic electrolysis cell with $Nd_2NiO_{4+\delta}$-based oxygen electrode, *Energ. Convers. Manage.* **2018,** *172*, 129-137, https://doi.org/10.1016/j.enconman.2018.07.014.
39. Liu, Z.; Cheng, D.; Zhu, Y.; Liang, M.; Yang, M.; Yang, G.; Ran, R.; Wang, W.; Zhou, W.; Shao, Z., Robust bifunctional phosphorus-doped perovskite oxygen electrode for reversible proton ceramic electrochemical cells, *Chem. Eng. J.* **2022,** *450*, 137787, https://doi.org/10.1016/j.cej.2022.137787.
40. Lyagaeva, J.; Danilov, N.; Vdovin, G.; Bu, J.; Medvedev, D.; Demin, A.; Tsiakaras, P., A new Dy-doped $BaCeO_3$–$BaZrO_3$ proton-conducting material as a promising electrolyte for reversible solid oxide fuel cells, *J. Mater. Chem. A* **2016,** *4* (40), 15390-15399, https://doi.org/10.1039/C6TA06414K.
41. Yu, X.; Ge, L.; Mi, Y.; Wu, B.; Yu, Z.; Jin, Z.; Zhao, Z.; He, B.; Chen, H.; Zheng, Y.; Cui, S., Superior Active and Durable Air Electrode for Protonic Ceramic Cells by Metal-Oxide Bond Engineering, *Small* **2025,** *21* (8), 2408607, https://doi.org/10.1002/smll.202408607.
42. He, F.; Liu, S.; Wu, T.; Yang, M.; Li, W.; Yang, G.; Zhu, F.; Zhang, H.; Pei, K.; Chen, Y.; Zhou, W.; Shao, Z., Catalytic self-assembled air electrode for highly active and durable reversible protonic ceramic electrochemical cells, *Adv. Funct. Mater.* **2022,** *32* (48), 2206756, https://doi.org/10.1002/adfm.202206756.

43. Zhang, L.; Li, X.; Zhang, L.; cai, H.; Xu, J.; Wang, L.; Long, W., Improved thermal expansion and electrochemical performance of $La_{0.4}Sr_{0.6}Co_{0.9}Sb_{0.1}O_{3-\delta}$-$Ce_{0.8}Sm_{0.2}O_{1.9}$ composite cathode for IT-SOFCs, *Solid State Sci.* **2019,** *91*, 126-132, https://doi.org/10.1016/j.solidstatesciences.2019.03.023.
44. Dyck, C. R.; Yu, Z. B. H.; Krstic, V. D., Thermal expansion matching of $Gd_{1-x}Sr_xCoO_{3-\delta}$ composite cathodes to $Ce_{0.8}Gd_{0.2}O_{1.95}$ IT-SOFC electrolytes, *Solid State Ionics* **2004,** *171* (1), 17-23, https://doi.org/10.1016/j.ssi.2004.02.014.
45. Liping, S.; Na, L.; Qiang, L.; Lihua, H.; Hui, Z., Preparation of $NdBaCo_2O_{5+\delta}$–$Ce_{0.9}Gd_{0.1}O_{1.95}$ composite cathode by in situ sol-mixing method and its high-temperature electrochemical properties, *J. Alloys Compd.* **2021,** *885*, 160901, https://doi.org/10.1016/j.jallcom.2021.160901.
46. Yao, C.; Zhang, H.; Liu, X.; Meng, J.; Zhang, X.; Meng, F.; Meng, J., Characterization of layered double perovskite $LaBa_{0.5}Sr_{0.25}Ca_{0.25}Co_2O_{5+\delta}$ as cathode material for intermediate-temperature solid oxide fuel cells, *J. Solid State Chem.* **2018,** *265*, 72-78, https://doi.org/10.1016/j.jssc.2018.05.028.
47. Yao, C.; Zhang, H.; Liu, X.; Meng, J.; Zhang, X.; Meng, F.; Meng, J., Investigation of layered perovskite $NdBa_{0.5}Sr_{0.25}Ca_{0.25}Co_2O_{5+\delta}$ as cathode for solid oxide fuel cells, *Ceram. Int.* **2018,** *44* (11), 12048-12054, https://doi.org/10.1016/j.ceramint.2018.03.206.
